# Direct reprogramming of human smooth muscle and vascular endothelial cells reveals defects associated with aging and Hutchinson-Gilford progeria syndrome

**DOI:** 10.7554/eLife.54383

**Published:** 2020-09-08

**Authors:** Simone Bersini, Roberta Schulte, Ling Huang, Hannah Tsai, Martin W Hetzer

**Affiliations:** 1 Molecular and Cell Biology Laboratory, The Salk Institute for Biological StudiesLa JollaUnited States; 2 Paul F. Glenn Center for Biology of Aging Research at The Salk InstituteLa JollaUnited States; 3 The Razavi Newman Integrative Genomics and Bioinformatics Core (IGC), The Salk Institute for Biological StudiesLa JollaUnited States; UT Health San AntonioUnited States; Weill Cornell MedicineUnited States

**Keywords:** smooth muscle cell, endothelial cell, direct reprogramming, aging, hutchinson-gilford progeria syndrome, vascular barrier, Human, Mouse

## Abstract

Vascular dysfunctions are a common feature of multiple age-related diseases. However, modeling healthy and pathological aging of the human vasculature represents an unresolved experimental challenge. Here, we generated induced vascular endothelial cells (iVECs) and smooth muscle cells (iSMCs) by direct reprogramming of healthy human fibroblasts from donors of different ages and Hutchinson-Gilford Progeria Syndrome (HGPS) patients. iVECs induced from old donors revealed upregulation of *GSTM1* and *PALD1*, genes linked to oxidative stress, inflammation and endothelial junction stability, as vascular aging markers. A functional assay performed on *PALD1* KD VECs demonstrated a recovery in vascular permeability. We found that iSMCs from HGPS donors overexpressed bone morphogenetic protein (*BMP*)−*4*, which plays a key role in both vascular calcification and endothelial barrier damage observed in HGPS. Strikingly, BMP4 concentrations are higher in serum from HGPS vs. age-matched mice. Furthermore, targeting BMP4 with blocking antibody recovered the functionality of the vascular barrier in vitro, hence representing a potential future therapeutic strategy to limit cardiovascular dysfunction in HGPS. These results show that iVECs and iSMCs retain disease-related signatures, allowing modeling of vascular aging and HGPS in vitro.

## Introduction

Physiological and pathological aging represent a major risk factor for the onset of cardiovascular diseases, the leading cause of death worldwide (Benjamin et al., 2019). The in vitro generation of human vascular cells (e.g. smooth muscle cells (SMCs), vascular endothelial cells (VECs)) represents a promising approach to regenerate compromised vascular beds (Leeper et al., 2010), as well as to model specific features of vascular dysfunction (e.g. blood brain barrier damage) (Vatine et al., 2019). Among the available strategies, direct reprogramming of skin fibroblasts into functional cells was previously reported for blood progenitor cells (Szabo et al., 2010), SMCs (van Tuyn et al., 2005) and VECs (Han et al., 2014; Morita et al., 2015). Direct reprogramming of cells from skin biopsies offers a critical advantage compared to other approaches such as the differentiation of induced pluripotent stem (iPS) cells. Indeed, although iPS cells have significantly contributed to the discovery of novel disease mechanisms (Park et al., 2019) as well as to the pre-clinical screening of promising drug candidates (Zhang et al., 2019), the differentiation process of iPS cells requires a mandatory transit through an embryonic-like state which was reported to reset the aging profile of the cells of origin (Mertens et al., 2015). Conversely, direct reprogramming from skin fibroblasts was shown to maintain the cellular signature of aging and to highlight critical factors of human aging in neurons (Mertens et al., 2015).

Previous studies reported that single or combined expression of specific transcription factors were able to directly reprogram skin fibroblasts into SMCs (Long et al., 2008) or VECs (Chen et al., 2019), which then expressed cell identity genes and were able to integrate within the mouse vasculature following subcutaneous or intramuscular implantation (Han et al., 2014; Morita et al., 2015). Strikingly, it was demonstrated that *ETV2* alone, which is a master regulator of VEC development and early vasculogenesis (Qiu and Hirschi, 2019), was sufficient to in vitro reprogram human skin fibroblasts into functional VECs (Morita et al., 2015). Similarly, the induced expression of myocardin (*MYOCD*) was able to strongly induce both smooth and cardiac muscle genes in human foreskin fibroblasts and mesenchymal stem cells (van Tuyn et al., 2005).

Since directly reprogrammed cells, but not iPS cells, preserve the aging signature of the donor (Mertens et al., 2015) and can be largely expanded from an easily obtained skin biopsy compared to other rare cell sources (e.g. blood-derived endothelial progenitor cells) (Mathur et al., 2019), we hypothesized that these cells could represent an effective tool to study cardiovascular aging and disease. In particular, Hutchinson-Gilford Progeria Syndrome (HGPS) is a rare age-related disease induced by mutation in the Lamin A/C gene (Prokocimer et al., 2013). This mutation not only alters the architecture of the nuclear envelope but also impacts the genome organization and gene expression leading to severe cardiovascular damage (e.g. atherosclerosis, vascular calcification), which is responsible for the premature death of patients at an average age of 14 years. In this scenario, the generation of directly reprogrammed vascular cells from HGPS patients would represent an invaluable tool to develop potential new therapies counteracting HGPS vascular degeneration, with the broad possibility to translate them for the treatment of vascular dysfunctions during physiological aging. Hence, the aim of this study was to generate, characterize, validate and use directly reprogrammed vascular cells, both induced VECs (i.e. iVECs) and induced SMCs (i.e. iSMC), to identify gene expression and functional differences between young and old individuals, as well as between healthy and HGPS donors. Our group has a strong expertise in the molecular and transcriptional characterization of human skin fibroblasts, which we recently combined with a machine learning approach to predict the biological aging of human donors (Fleischer et al., 2018). Based on these premises, here we combined RNAseq and functional studies of vascular permeability in 3D microscale models which allowed us to identify potential new biomarkers of vascular aging and to explore the pathological cross-talk between SMCs and VECs in HGPS patients.

## Results

### iSMCs and iVECs express cell-identity markers and form 3D vascular structures in vitro

Direct reprogramming of skin fibroblasts into tissue-specific cells (e.g. neurons, vascular cells, blood cells) is a relatively new research field which can potentially affect both tissue engineering applications and in vitro disease modeling. Recently, several attempts have been made to generate reprogrammed vascular cells (Han et al., 2014; van Tuyn et al., 2005), but a complete characterization of their gene expression profile compared to the original fibroblast population, as well as to primary vascular cells, is still missing.

**Figure 1.**
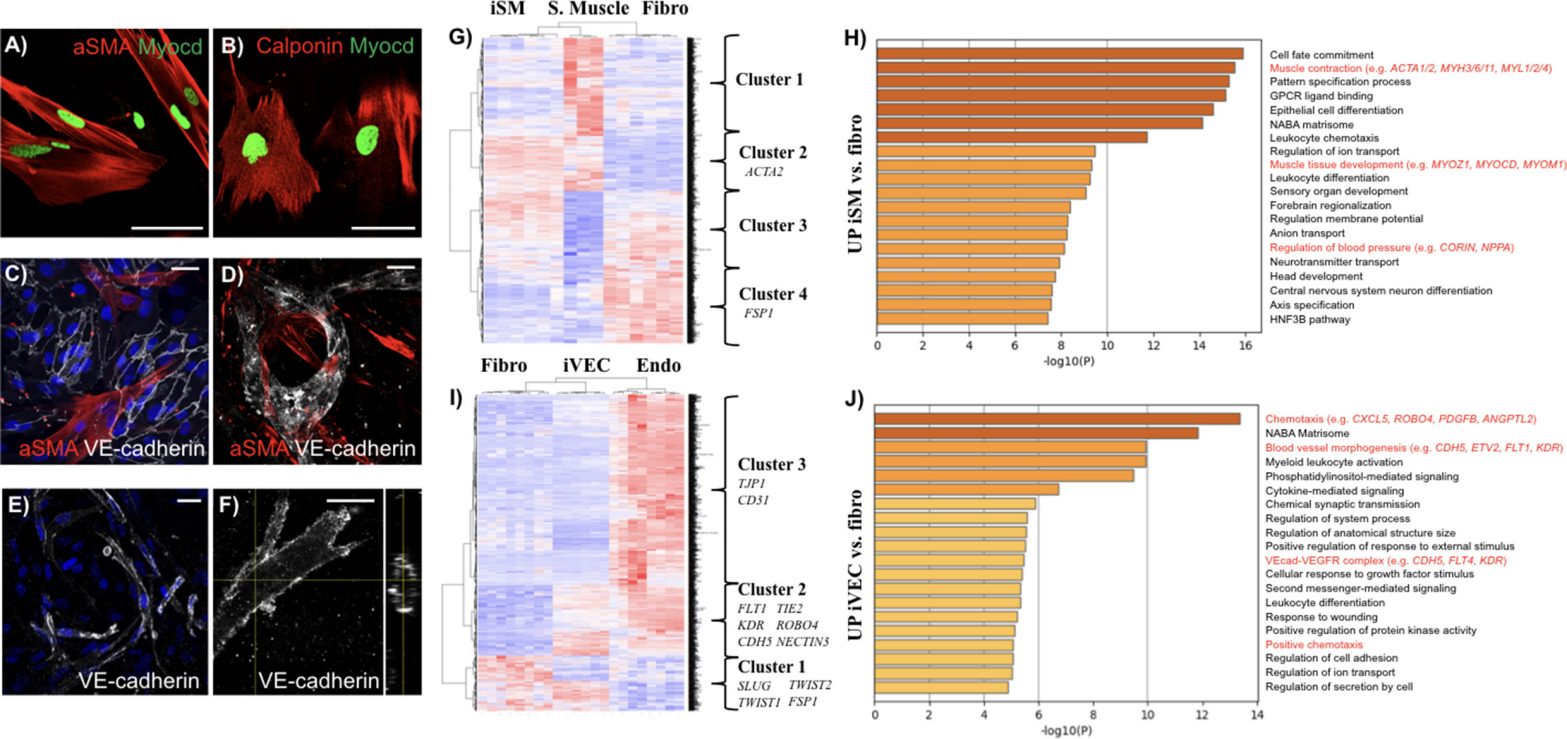
Characterization of directly reprogrammed vascular cells. iSMCs express the identity markers αSMA (**A**) and calponin (**B**). iSMCs contribute to the formation of endothelial monolayers (**C**) and 3D microvascular networks (**D**). iVECs form 3D vessel-like structures in fibrin matrix (**E**) and show open lumens (**F**). Both iSMCs and iVECs express cell identity genes showing a progressive transition from fibroblasts to mature vascular cells (**G–J**). iSMC gene expression profile compared to primary SMCs and fibroblasts (analysis based on 1,852 DE genes between primary SMCs and fibroblasts). N = 6 donors for iSMCs and fibroblasts, N = 3 donors for SMCs. Z score = ± 3. DE genes with log_2_FC > 1 and FDR < 0.05 (**G**). Gene ontology analysis reveals that iSMCs upregulate genes associated with muscle development, contraction and blood pressure regulation compared to fibroblasts (**H**). iVEC gene expression profile compared to primary VECs and fibroblasts (analysis based on 1,780 DE genes between primary VECs and fibroblasts). N = 6 donors for iVECs, primary VECs and fibroblasts. Z score = ± 4. DE genes with log_2_FC > 1 and FDR < 0.05 (**I**). Gene ontology showing that iVECs upregulate genes associated with chemotaxis, blood vessel morphogenesis and VE cadherin–VEGFR complex compared to fibroblasts, indication of ongoing differentiation towards and endothelial phenotype (**J**). Scale bars: 25 µm. Figure 1—source code 1.Statistical models used for DE analysis and clustering analysis. Figure 1—source data 1.DE analysis. Figure 1—source data 2.Clustering analysis.

Since SMCs and VECs play an important role in human aging and HGPS (Hamczyk et al., 2019; Li et al., 2018), we directly induced iSMCs and iVECs by isolating skin fibroblasts from human donors and overexpressing either the master regulators *MYOCD* or *ETV2*, respectively. iSMCs expressed cell-identity markers including alpha smooth muscle actin (αSMA) (Figure 1A, expressed by about 70% cells) and calponin (Figure 1B, expressed by about 80% cells), both in monoculture and when co-cultured with VECs (Figure 1C). Importantly, αSMA-expressing iSMCs contributed to the formation of microvascular networks in 3D fibrin matrices (Figure 1D), thus mimicking the behavior of fibroblasts and differentiated mesenchymal stem cells observed in previous vascular models developed by our (Bersini et al., 2016; Jeon et al., 2014) and other groups (Zheng et al., 2012). In particular, no differences were found in terms of vascular area fraction when VECs were co-cultured with fibroblasts or iSMCs (Figure 1—figure supplement 1). In parallel, we found that CD31+ iVECs (Figure 1—figure supplement 2) were able to self-assemble into vessel-like structures when co-cultured with skin fibroblasts (Figure 1E) or iSMCs (Video 1) in 3D fibrin matrices. These vessel-like structures were patent, as demonstrated by cross-sectional views of the lumens (Figure 1F).

**Figure 1—figure supplement 1.**
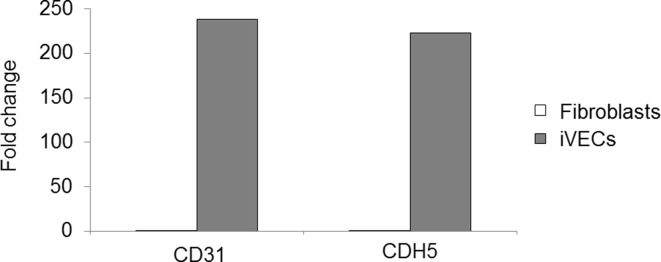
Quantification of the vascular area fraction in 3D matrices embedding a co-culture of VECs and fibroblasts or iSMCs (Student’s t-test with p=0.61).

**Figure 1—figure supplement 2.**
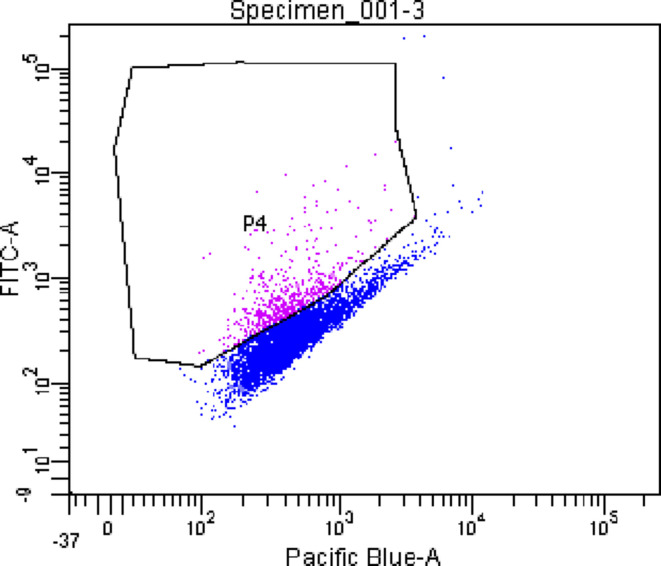
Representative FACS plot showing iVEC sorting based on CD31 expression. Region P4 refers to CD31 positive cells labeled with AlexaFluor488 secondary antibody.

**Video 1.** Representative movie showing a 3D reconstruction of in vitro co-cultured iVECs (white) and iSMCs (red) in a fibrin matrix.

To further define the reprogramming status of iSMCs and iVECs, we performed RNAseq analysis on these induced cells and compared them with the donor fibroblast population as well as with human primary smooth muscle and endothelial cell lines. Specifically, we initially compared the expression profile of skin fibroblasts (N = 6 donors) vs. primary SMCs (N = 3 donors, data obtained from GEO) or skin fibroblasts (N = 6 donors) vs. primary VECs (N = 6 donors, data generated for this study using commercially available skin microvascular endothelial cells) (Supplementary file 1). This analysis allowed us to identify about 1800 differentially expressed (DE) genes in both comparisons, which defined the identity of fibroblasts, SMCs and VECs (Supplementary file 2, 3 and 4). Then, we used these DE genes as a benchmark to analyze the gene expression of iSMCs (Figure 1G and H) and iVECs (Figure 1I and J). The comparison among skin fibroblasts, primary vascular cells and reprogrammed cells highlighted multiple gene clusters representative of specific gene expression signatures. Strikingly, we found that the SMC identity gene *ACTA2* (Figure 1G, cluster 2) and multiple endothelial-specific genes (e.g. *FLT1, KDR, CDH5, TIE2, ROBO4, NECTIN2*) (Figure 1I, cluster 2) were enriched in both reprogrammed cells and primary vascular cells when compared to the original skin fibroblasts. As typical for induced cells, they did not express all cell identity markers of primary vascular cells. For example, iVECs expressed less *CD31* and *TJP1* compared to primary endothelial cells (Figure 1I, cluster 3). However, the expression level of *KDR* (an important gene defining endothelial cell identity) was 6.62 log_2_FoldChange (FC) higher in primary VECs vs. fibroblasts and 6.38 log_2_FC higher in iVECs vs. fibroblasts. At the same time, another endothelial-specific gene (i.e. *CDH5*) was upregulated in both primary cells (log_2_FC = 6.7) and iVECs (log_2_FC = 4) compared to fibroblasts, although at different levels (Supplementary file 5). Similarly, both primary and reprogrammed SMCs upregulated key identity genes including *ACTA2* and *MYH10* compared to fibroblasts (Supplementary file 5). Notably, the expression of *MYH10* was lower in reprogrammed vs. primary SMCs. Furthermore, key skin fibroblast cell identity genes (e.g. *FSP1*, *SLUG, TWIST1, TWIST2*) (Figure 1G, cluster 4; Figure 1I, cluster 1) were downregulated in both reprogrammed and primary vascular cells (Supplementary file 5).Finally, gene ontology analysis revealed that iVECs upregulated genes linked to blood vessel morphogenesis and VE-cadherin-VEGF receptor complex when compared to fibroblasts (Figure 1J), while iSMCs upregulated genes associated with muscle contraction and muscle development (Figure 1H).

Taken together, the majority of the markers expressed by reprogrammed cells remained fibroblast-like. After carefully considering this critical point, we would like to highlight that reprogrammed SMCs and VECs expressed typical cell identity genes, self-organized into 3D vascular structures mimicking simple vascular networks and showed a global gene expression signature representing a gradual transition towards fully mature cells.

### Induced vascular cells show gene-expression and functional differences between young vs. old donors

**Figure 2.**
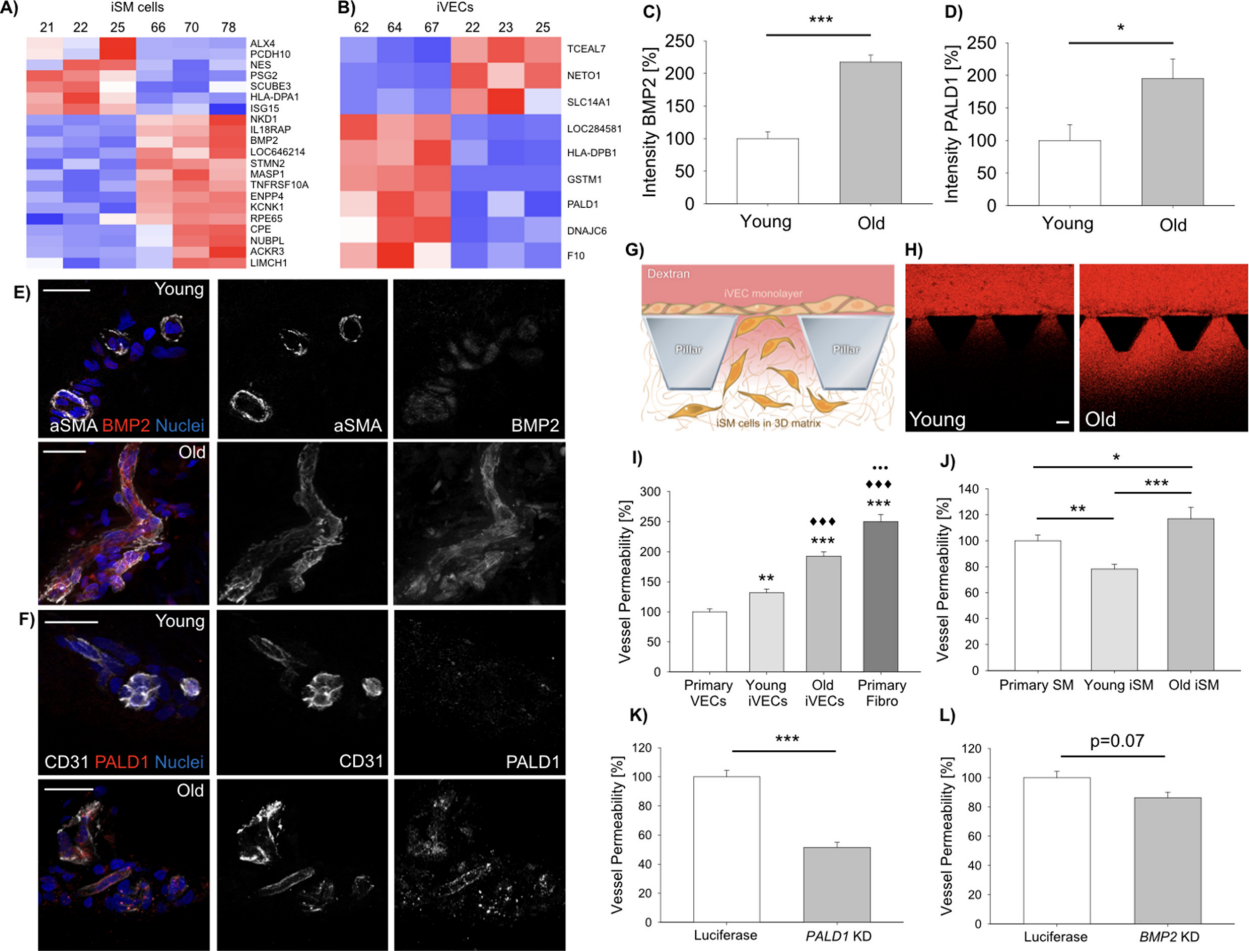
Vascular cells reprogrammed from young vs. old donors show gene expression and functional differences. Heat-map representing DE genes between reprogrammed vascular cells from young (N = 3) vs. old (N = 3) donors (iSMCs (**A**), iVECs (**B**)). Z score = ± 1.5. DE genes with log_2_FC > 1 and FDR < 0.05. Quantification of BMP2 (**C**) and PALD1 (**D**) expression in human skin biopsies from young vs. old donors (N = 2 donors per condition; N = 10 tissue sections per condition; Student’s t-test with p<0.001 (***) and p<0.05 (*)). Representative images of BMP2 (**E**) and PALD1 (**F**) from skin biopsies obtained from young vs. old donors. Scale bars: 50 µm. Quantification of vascular permeability using young vs. old reprogrammed cells (**G–J**). Schematic showing the generation of an endothelial monolayer covering the interface with a 3D matrix embedding SMCs. Vascular permeability was quantified by measuring the variation of 70 kDa dextran fluorescent intensity across the interface (**G**). Representative images of dextran flow (red) through the endothelial monolayer in presence of young vs. old reprogrammed cells. Scale bar: 100 µm (**H**). Quantification of vascular permeability in presence of young vs. old iVECs (I, at least N = 40 independent measurements per condition in three biological replicates; ANOVA with Holm-Sidak test; comparison with primary VECs (** is p<0.01 and *** is p<0.001); comparison with young iVECs (♦♦♦is p<0.001); comparison with old iVECs (••• is p<0.001)) or young vs. old iSMCs (J, at least N = 40 independent measurements per condition in three biological replicates; ANOVA with Holm-Sidak test; * is p<0.05, ** is p<0.01 and *** is p<0.001). Quantification of vascular permeability in presence of *PALD1* KD (**K**) or *BMP2* KD (**L**) in primary VECs and SMCs, respectively (at least N = 60 independent measurements per condition in three biological replicates for each KD experiment; Student's t-test; *** is p<0.001).

Previous studies demonstrated that directly reprogrammed, but not iPS cell-derived neurons, were able to retain the transcriptional aging signature of the donor (Mertens et al., 2015). We therefore asked if iSMCs and iVECs could be employed to identify genes involved in the vascular degeneration occurring during physiological aging (Ungvari et al., 2018). Aging is characterized by a progressive decrease in the functionality of the endothelial barrier (Palmer and Ousman, 2018), formation of atherosclerotic lesions, reduced contractility and calcifications of SMCs (Yao et al., 2013). Hence, we specifically aimed at identifying genes potentially involved in these processes. To answer this question, we established fibroblast cell lines from skin biopsies of N = 3 young (19–30 y.o.) and N = 3 old (62–87 y.o.) healthy donors (Supplementary file 1) and directly reprogrammed them into iSMCs or iVECs using the master regulators *MYOCD* and *ETV2*, respectively. It is important to highlight that the expression of *MYOCD* and *ETV2* was comparable in reprogrammed cells derived from young and old donors (log_2_FC = 0.03 for *MYOCD* and log_2_FC = 0.19 for *ETV2* comparing young and old cells; no statistical difference comparing young and old cells). DE analysis highlighted a set of genes (21 DE genes for iSMCs; 9 DE genes for iVECs) that changed according to the age of the donor for either iSMCs (Figure 2A) or iVECs (Figure 2B). We then analyzed the expression of a few cell identity genes in iSMCs or iVECs derived from young and old donors without finding obvious age-related differences (Supplementary file 6 and Figure 2—figure supplement 1). Together with the similar expression of MYOCD and ETV2 in cells derived from young and old donors, these data suggest that the reprogramming efficiency should not be affected by the age of the donor and that potential differences between young and old cells do not seem to be due to different levels of reprogramming.

**Figure 2—figure supplement 1.**
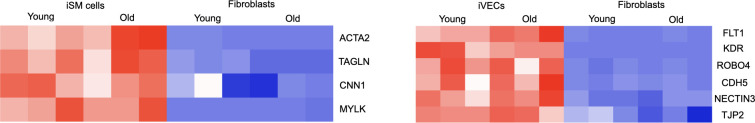
Heat-maps representing key cell identity genes for both iSM cells and iVECs derived from young and old donors. Fibroblasts were included as control. Z score = ± 1. All these genes were not DE comparing young and old donors.

**Figure 2—figure supplement 2.**
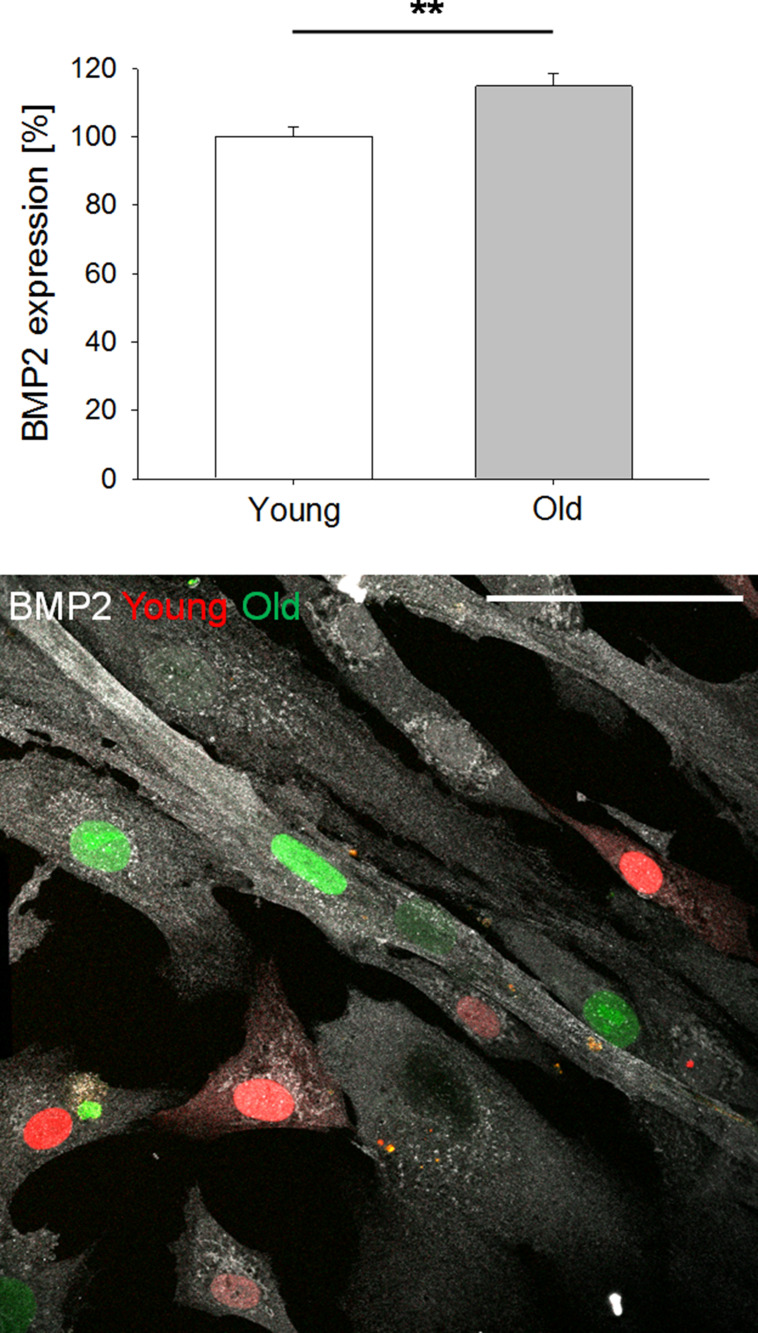
BMP2 expression by iSMCs reprogrammed from young (19 y.o.) vs. old (67 y.o.) donor. Representative image showing young (mCherry MYOCD) and old (EGFP MYOCD) iSMCs. Experiments were also performed with the reciprocal tagging. At least N = 25 independent measurements in three biological replicates. Student’s t-test, p<0.01 (**). Scale bar: 50 µm.

**Figure 2—figure supplement 3.**
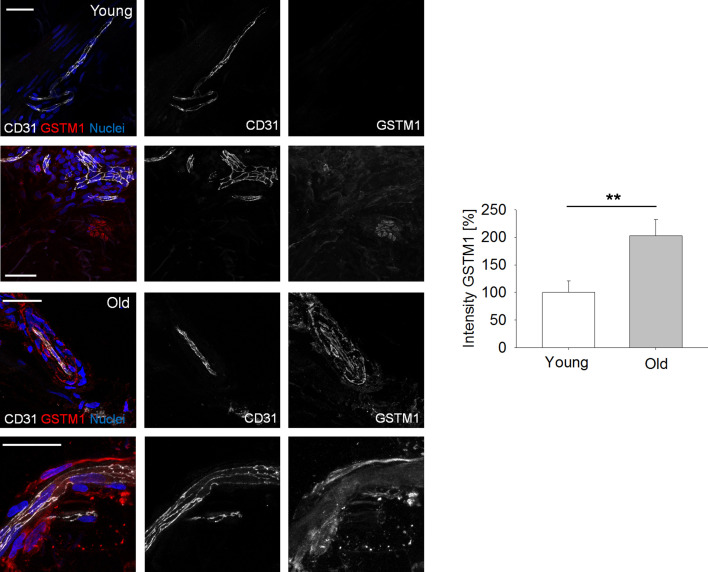
Representative images of GSTM1 expression by endothelial cells within human skin biopsies from young vs.old donors (N = 2 donors per condition, 10 tissue sections per condition; Student’s t-test with p<0.01 (**)). Scale bars: 100 µm.

**Figure 2—figure supplement 4.**
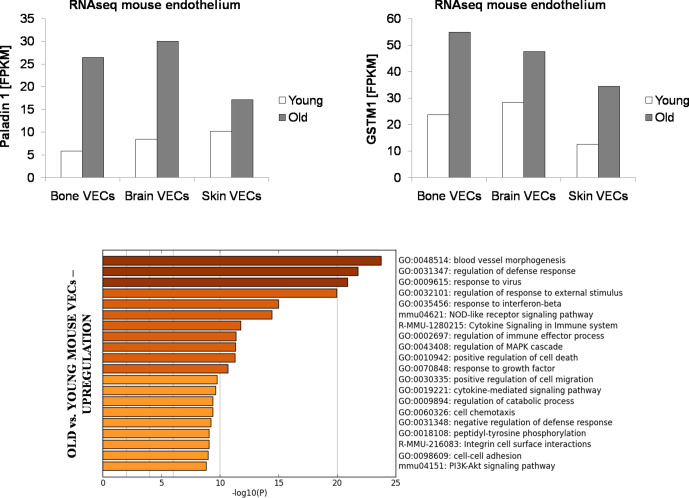
FPKM values for *PALD1*and *GSTM1* from RNAseq performed on primary mouse endothelial cells from bone marrow, brain and skin of young (3 months) vs. old (18 months) mice. Biological processes upregulated in old vs. young mouse VECs (pool of bone marrow, brain and skin VECs). It is interesting to observe the upregulation in old mouse VECs of biological processes associated with immune system activation and inflammation, consistent with the general concept that aging is associated with chronic inflammation. Cells were derived from N = 3 animals per age group and then pooled for RNAseq.

**Figure 2—figure supplement 5.**
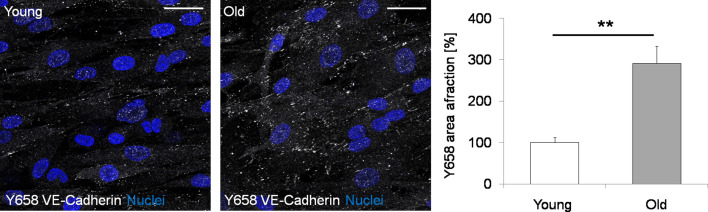
Representative images and quantification of Y658 VE-Cadherin (white) in iVECs derived from young and old donors. The higher amount of Y658 staining in old iVECs (Student’s t-test with p<0.01 (**) from N = 3 biological replicates) suggests that cell-cell junctions might be compromised in reprogrammed cells derived from old donors. Nuclei: blue. Scale bars: 25 µm.

**Figure 2—figure supplement 6.**
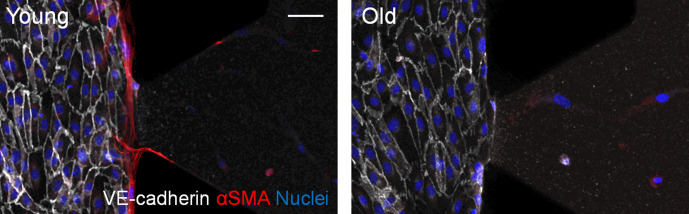
Representative images of microfluidic devices seeded with iSMCs reprogrammed from young or old fibroblasts. VECs stained with VE-cadherin (white) cover the lateral channel and the interface with the 3D matrix embedding iSMCs (red, αSMA). iSMCs from young donors migrate towards the endothelial layer. Nuclei: blue. Scale bar: 50 µm.

**Figure 2—figure supplement 7.**
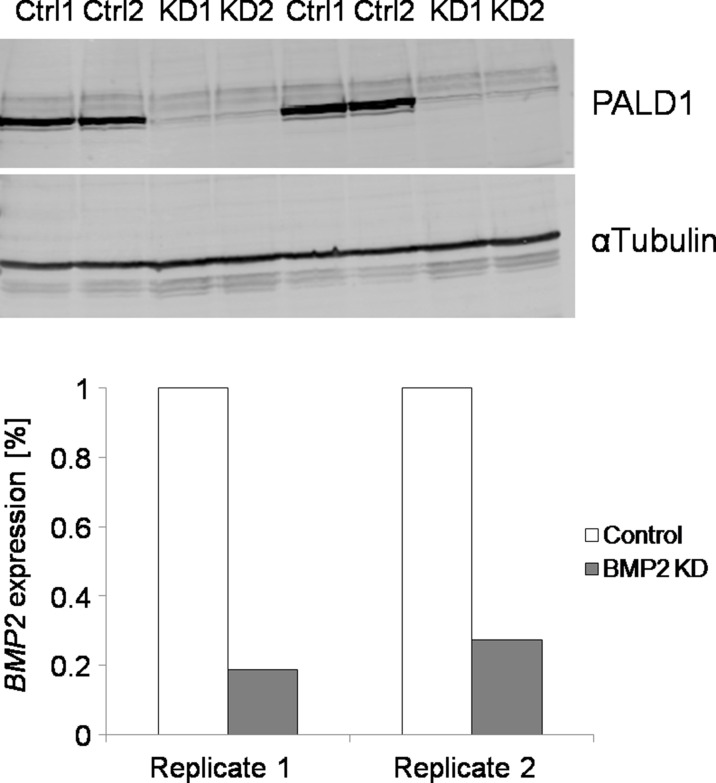
Validation of KD of *PALD1* and *BMP2* in VECs and SMCs, respectively (N = 2 biological replicates). Note that *BMP2* KD was validated using qPCR due to technical problems with any of the tested anti-BMP2 antibodies (Western Blot). The use of these antibodies resulted in multiple aspecific bands.

Intriguingly, among the 21 DE genes, iSMCs from older donors showed upregulation of bone morphogenetic protein 2 (*BMP2)*, which is involved in the vascular calcification of blood vessels during aging and disease (Petsophonsakul et al., 2019). Significantly, we found that αSMA+ cells in human skin biopsies from old (N = 2) vs. young (N = 2) donors expressed higher levels of BMP2 (217.4 ± 10.9% vs. 100.0±10.4% after normalization, Figure 2C and E; Videos 2 and 3), in agreement with the RNAseq findings. Noteworthy, the pattern of αSMA+ cells suggests that these cells are actually surrounding blood vessels rather than being isolated in the skin extracellular matrix (Figure 2E). Finally, we co-cultured iSMCs generated from a young (19 y.o.) and an old (67 y.o.) donor finding higher expression of BMP2 in the iSMCs derived from the old donor (Figure 2—figure supplement 2), confirming observations seen with RNAseq and immunofluorescence of skin biopsies.

**Video 2.** Representative movie showing a 3D reconstruction of human skin biopsy from a young (21 y.o.) donor stained for BMP2. From the left: panel one is BMP2, panel two is αSMA, panel three is DAPI and panel four is merge.

**Video 3.** Representative movie showing a 3D reconstruction of human skin biopsy from an old (72 y.o.) donor stained for BMP2. From the left: panel one is BMP2, panel two is αSMA, panel three is DAPI and panel four is merge.

In parallel, DE analysis performed on iVECs highlighted the upregulation of *GSTM1* and *PALD1* in cells derived from old vs. young donors. *GSTM1* is generally activated in presence of oxidative stress and inflammatory conditions (Lopes-Aguiar et al., 2017), while *PALD1* is an endothelial-specific gene inhibiting endothelial junction stability (Nitzsche, 2016). To test if these genes were actually dysregulated in human donors, we analyzed human skin biopsies and found that CD31+ VECs from old vs. young healthy donors indeed expressed higher levels of PALD1 (194.9 ± 30.0% vs. 100.0±24.3% after normalization, Figure 2D and F; Videos 4 and 5) and GSTM1 (Figure 2—figure supplement 3). This result shows that the gene expression differences observed in directly reprogrammed cells partially recapitulate signatures of aging in humans. Strikingly, DE analysis on primary VECs from mouse skin, brain and bone marrow also highlighted a clear upregulation of *GSTM1* and *PALD1* in cells obtained from old (18 months) vs. young (3 months) animals (N = 3 animals per condition, Figure 2—figure supplement 4). Together, these data suggest that both PALD1 and GSTM1 might represent evolutionary conserved vascular aging markers.

**Video 4.** Representative movie showing a 3D reconstruction of human skin biopsy from a young (21 y.o.) donor stained for Paladin 1. From the left: panel one is Paladin, panel two is CD31, panel three is DAPI and panel four is merge.

**Video 5.** Representative movie showing a 3D reconstruction of human skin biopsy from an old (72 y.o.) donor stained for Paladin 1. From the left: panel one is Paladin, panel two is CD31, panel three is DAPI and panel four is merge.

Next we wanted to test if these aging markers were functionally and mechanistically linked to vascular aging. Since abnormal expression of BMPs has been linked to vascular dysfunction (Cai et al., 2012; Csiszar et al., 2009) and upregulation of PALD1 to compromised VEC junctions (Nitzsche, 2016), we hypothesized that the identified DE genes might reflect a functional change in vascular permeability. To test this directly, we employed an in vitro 3D model where VECs lining a microfluidic channel form a continuous barrier separating the channel from a 3D fibrin matrix embedding SMCs (Figure 2G and H). The presence of old vs. young iVECs (N = 3 donors per condition) lining the interface with the matrix channel increased the vascular permeability (146.1 ± 5.4% vs. 100.0±4.6% after normalization for old vs. young iVECs). The difference in permeability can be explained by partial damage to cell-cell junctions, as indicated by the different pattern of Y658 VE-Cadherin in old vs. young iVECs (Figure 2—figure supplement 5). Comparison with positive control (primary VECs lining the channel) and negative control (fibroblasts lining the channel) revealed that permeability of young (131.9 ± 6.1%) and old (192.7 ± 7.1%) iVECs was significantly lower compared to fibroblasts (250.0 ± 12.1%), reaching values close to primary VECs (100.0 ± 4.7%) (Figure 2I, data normalized to the permeability of primary VECs). Similarly, the presence of old vs. young iSMCs (N = 3 donors per condition) in the fibrin matrix channel induced a significant increase in the leakiness of the endothelial barrier (149.8 ± 11.3% vs. 100.0±4.7%, data normalized to young iSMCs). iSMCs from young donors induced even lower levels of vascular permeability compared to primary SMCs (78.2 ± 3.7% vs. 100.0 ± 4.3%) (Figure 2J, data normalized to primary SMCs), demonstrating that direct reprogramming generates functional cells which contribute to the maintenance of the vascular barrier. Surprisingly, we also found that iSMCs from young vs. old donors were more migratory, being able to reach the endothelial barrier at the interface with the 3D matrix (Figure 2—figure supplement 6). These findings are not only in agreement with in vivo observations but directly link iSMCs to the appearance of functional and morphological changes during aging.

To test the functional significance of two of the identified DE genes that might play a role in vascular permeability (i.e. *PALD1* and *BMP2*), we performed knockdown (KD) experiments on VECs and SMCs (Figure 2—figure supplement 7). Collected data showed that *PALD1* KD in VECs decreased vascular permeability compared to luciferase control (51.5 ± 3.4% vs. 100.0 ± 4.3%) (Figure 2K, data normalized to the permeability with luciferase control). On the other side, KD of *BMP2* in SMCs induced a weak, but reproducible, decrease in permeability compared to luciferase control (86.3 ± 3.8% vs. 100.0 ± 4.3%) (Figure 2L, data normalized to luciferase control).

Overall, while we do not claim that reprogrammed cells are transcriptionally and functionally identical to primary cells, these data clearly demonstrate that reprogramming induces a minimum set of transcriptional and functional features which allow to capture a fraction of the vascular changes occurring during aging and disease. This aging signature could be in principle used to longitudinally identify features of vascular degeneration with minimally invasive skin biopsies.

### Induced vascular cells recapitulate hallmarks of vascular dysfunction of HGPS patients

HGPS is a rare form of accelerated aging due to mutation in the Lamin A/C gene. Patients exhibit severe cardiovascular damage which leads to premature death (Stehbens et al., 2001). In particular, HGPS is characterized by dysfunctional SMCs. Phenotypic changes in SMCs are often associated with vascular calcification (Wei et al., 2018) and hypertension (Miriyala et al., 2006) in a broad spectrum of diseases. For these reasons we sought to determine if iSMCs directly reprogrammed from HGPS vs. healthy fibroblasts could retain a signature of vascular dysfunction, with the ultimate goal to identify new potential therapies to slow down the process of vascular degeneration.

**Figure 3.**
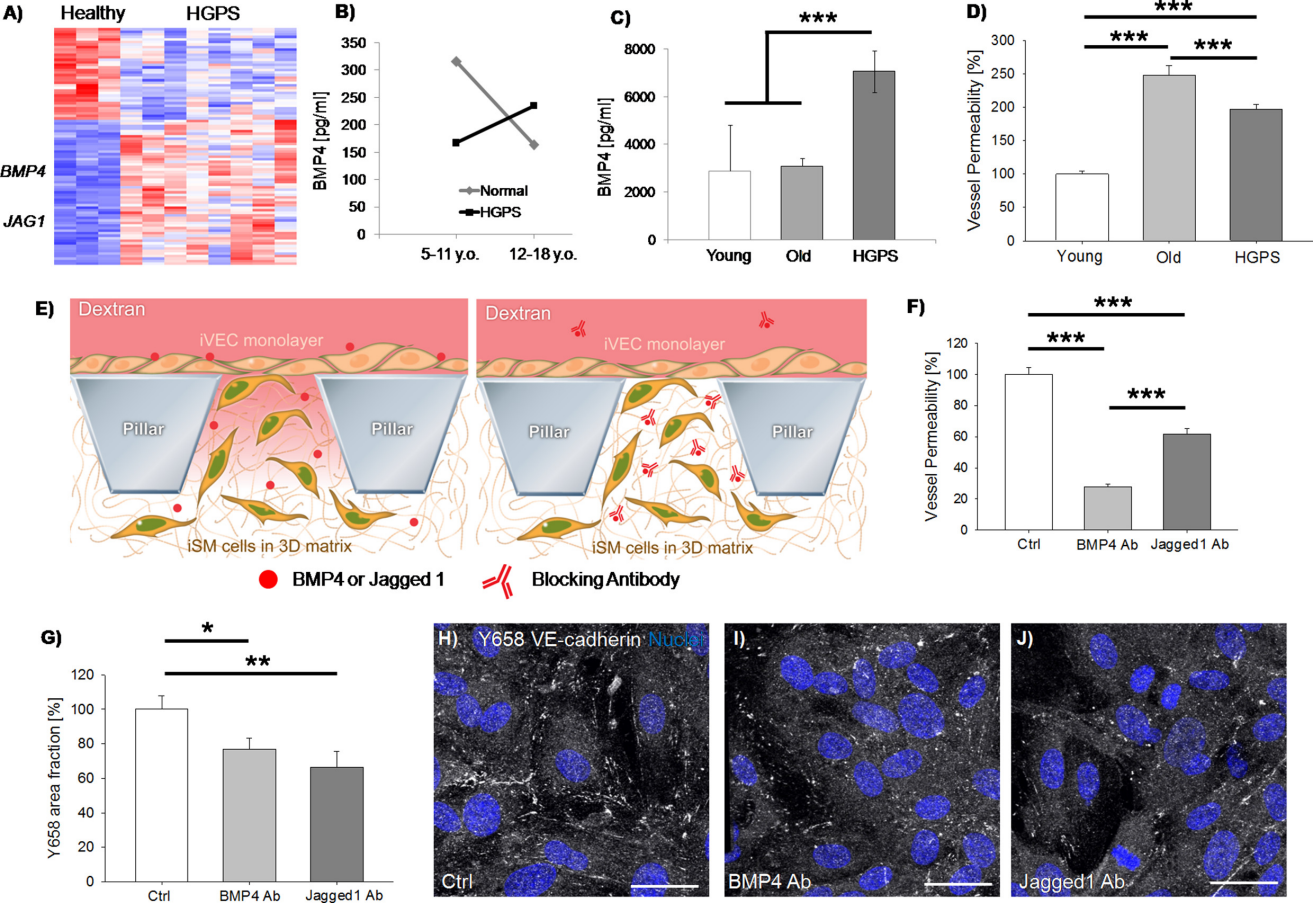
iSMCs reprogrammed from HGPS fibroblasts show signatures of vascular dysfunction. Heat-map representing DE genes between iSMCs reprogrammed from healthy (N = 3) vs. HGPS (N = 8) donors. Z score = ± 2. DE genes with log_2_FC > 1 and FDR < 0.05 (**A**). ELISA measurement of BMP4 in the serum of HGPS and age-matched healthy human donors (B, Student’s t-test with p=0.4 for normal vs. HGPS 5–11 y.o.; p=0.575 for normal vs. HGPS 12–18 y.o.; Student’s t-test with p=0.04 for 5–11 y.o. vs. 12–18 y.o. normal donors; Student’s t-test with p=0.4 for 5–11 y.o. vs. 12–18 y.o. HGPS donors), as well as in the serum of HGPS, young (age-matched) and old mice (C, ANOVA with Holm-Sidak test with p=0.79 for young vs. old; p=0.001 for young vs. HGPS; p<0.001 for old vs. HGPS). Quantification of vascular permeability in presence of iSMCs reprogrammed from young (N = 6), old (N = 6) and HGPS (N = 6) donors. At least N = 95 independent measurements per condition in three biological replicates. ANOVA with Holm-Sidak test with p<0.001 (***) (**D**). Schematic showing the vascular permeability assay in presence of antibodies specifically blocking secreted BMP4 and Jagged 1 (**E**). Quantification of vascular permeability comparing iSMCs from HGPS donors (ctrl) or the same cells treated with antibodies blocking BMP4 or Jagged 1. At least N = 60 independent measurements per condition in three biological replicates. ANOVA with Holm-Sidak test with p<0.001 (***) (**F**). Quantification of the effect of BMP4 and Jagged one blocking antibody on Y658 VE-cadherin area fraction. N = 6 independent measurements per condition in three biological replicates. ANOVA with Holm-Sidak test with p<0.05 (*) or p<0.01 (**); p=0.897 for BMP4 Ab vs. Jagged1 Ab (**G**). Representative images of Y658 VE-cadherin in presence of BMP4 and Jagged one blocking antibodies (**H–J**). Scale bars: 25 µm.

Following the same strategy used to identify signatures of vascular aging, we performed RNAseq on iSMCs derived from N = 3 healthy donors vs. N = 8 HGPS donors (Supplementary file 1) revealing 93 DE genes (Figure 3A). Among the identified DE genes, we found upregulation of *BMP4*, which is generally overexpressed during the early onset of vascular calcification (Wei et al., 2018). Moreover, secreted BMP4 enhances the activation of the endothelium and induces production of reactive oxygen species by VECs (Lowery and de Caestecker, 2010), compromising the vascular barrier. To test the clinical significance of BMP4, we obtained human blood serum from HGPS and age-matched healthy controls (N = 7 donors per group) from the Progeria Research Foundation. Interestingly, we found that BMP4 was higher in healthy vs. HGPS donors below 11 years old, while a reverse trend was observed for children above 12 years old (Figure 3B). Analyzing these data from an aging perspective, we found a significant decrease in the level of BMP4 in older vs. younger healthy individuals (p=0.04) which was lost in the HGPS samples. This abnormal trend suggests that a potential correlation might exist between the vascular damage in HGPS patients and circulating BMP4. Indeed, it is reported that the level of BMP4 is high during early development, being a key regulator of musculo-skeletal system formation and vascularization. BMP4 decreases during late childhood/young adulthood and then increases again during physiological aging (Meyers et al., 2016), as well as in pathological conditions including metabolic syndrome (Son et al., 2011), leukemia (Voeltzel et al., 2018) or bone fractures (van Baardewijk et al., 2013). Given the extremely limited number of available samples from HGPS patients, we then collected blood from HGPS mouse models carrying the progerin mutation G609G (N = 3, 13–14 weeks old) and we compared the level of circulating BMP4 with age-matched healthy mice (N = 3, 15 weeks old) as well as with extremely old mice (N = 5, about 2 years old). Collected data revealed a higher level of BMP4 in the blood of HGPS mice (Figure 3C), hence further corroborating the analyses performed on human samples.

**Figure 3—figure supplement 1.**
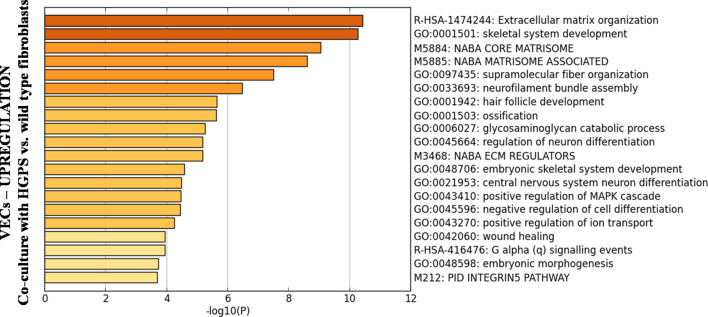
Biological processes upregulated in primary, healthy VECs when 3D co-cultured with HGPS vs. healthy fibroblasts for 25 days. These supplementary data refer to N = 1 biological replicate.

Multiple dysregulated pathways contribute to the age-related and pathological vascular remodeling observed in human patients. In this context, the Jagged 1-Notch pathway plays a pivotal role. Indeed, recent studies demonstrated that Jagged 1-mediated Notch activation induces hyper-permeability of blood vessels in a model of diabetic retinopathy and that blocking Jagged 1 can prevent retinal edema (Miloudi et al., 2019). Intriguingly, we found that iSMCs from HGPS vs. healthy donors upregulated *JAG1*, suggesting its potential contribution in driving the pathological remodeling of blood vessels in HGPS patients.

Based on these findings, we hypothesized that the identified DE genes in reprogrammed iSMCs from HGPS vs. healthy donors could partially explain potential changes in the vascular barrier functionality. To test this hypothesis, we employed microfluidic devices embedding iSMCs reprogrammed from fibroblasts of young, old or HGPS donors (N = 6 donors per group) in co-culture with old VECs. Strikingly, we found that the presence of HGPS iSMCs determined a significant increase in the vascular leakiness compared to young donors (197.4 ± 7.5% vs. 100.0±4.6% after normalization, Figure 3D).

To verify that BMP4 and Jagged 1 were actually playing a key role in the regulation of vascular permeability, we treated iSMCs from HGPS donors with antibodies blocking secreted BMP4 or Jagged 1 (Figure 3E). Strikingly, we found a significant decrease in the vascular leakiness upon antibody treatment in both conditions compared to control (27.8 ± 1.6% vs. 100.0 ± 4.4% for BMP4 antibody after normalization and 61.5 ± 3.8% vs. 100.0 ± 4.4% for Jagged 1 antibody after normalization; Figure 3F and Videos 6, 7 and 8). Noteworthy, BMP4 antibody was able to reduce the vascular permeability to a value even lower than the level detected in presence of iSMCs from young donors (indeed we quantified 72.2% decrease in vascular permeability with BMP4 antibody-treated HGPS cells and only 49.3% decrease comparing iSMCs from young vs. HGPS donors). Immunofluorescence staining of the endothelial cell-cell junction marker VE-cadherin did not show any macroscopic difference between HGPS iSMCs with and without BMP4/Jagged 1 blocking antibody. However, we did observe an increase in the amount of Y658 VE-cadherin (indication of cell-cell junction destabilization) in presence of untreated iSMCs, in agreement with the higher vascular leakiness (Figure 3G–J).

**Video 6.** Representative movie showing 70 kDa dextran diffusion through the endothelial barrier in presence of iSMCs reprogrammed from HGPS donors.

**Video 7.** Representative movie showing 70 kDa dextran diffusion through the endothelial barrier in presence of iSMCs reprogrammed from HGPS donors and treated with BMP4 blocking antibody.

**Video 8.** Representative movie showing 70 kDa dextran diffusion through the endothelial barrier in presence of iSMCs reprogrammed from HGPS donors and treated with Jagged 1 blocking antibody.

Surprisingly, we also found that primary, healthy VECs upregulated *BMP4* and other genes associated with calcification, skeletal system development and extracellular matrix organization when co-cultured with HGPS fibroblasts (Figure 3—figure supplement 1, N = 1 biological replicate).

Overall, using our validated approach we identified two previously unexplored target proteins which are secreted by HGPS SMCs and directly affect the functionality of the vascular barrier. These secreted proteins can be targeted through blocking antibodies to revert the phenotype of VECs, suggesting a potential application to restore the barrier function in HGPS patients.

## Discussion

We have described a powerful strategy to study vascular aging and human pathologies affecting the cardiovascular system through directly reprogrammed vascular cells. To validate our strategy, we have provided to our knowledge the most complete characterization of reprogrammed vascular cells by comparing them with the skin fibroblasts from which they are derived and with primary vascular cells. While both iSMCs and iVECs express key cell identity markers and contribute to the formation of in vitro 3D vascular structures, their degree of maturation does not allow ta claim of a complete replica of their primary counterparts. Indeed, most of the markers expressed by reprogrammed cells remained fibroblast-like and all the results here presented should be viewed cwith this point in mind. Nevertheless, reprogrammed cells showed indications of progressive transition from the fibroblast to the vascular lineage considering a small set of cell identity genes. These cells could then be successfully employed to identify genes involved in the vascular degeneration observed during physiological aging. Despite the limited number of DE genes comparing reprogrammed cells from young vs. old donors, the identified targets reflect typical changes expected during vascular aging, as demonstrated by their validation within human skin biopsies. While we were surprised for the low number of identified DE genes between young and old donors, this observation is consistent with other studies comparing cells from donors of different biological age (Fleischer et al., 2018; Mertens et al., 2015).

Strikingly, we found that reprogrammed cells from young vs. old donors were also characterized by functional differences, as demonstrated by the compromised barrier function in presence of iSMCs or iVECs from old individuals. We noticed that the levels of permeability were about 50% higher in presence of reprogrammed cells from old vs. young donors. The observed difference in permeability seems to reflect intrinsic differences between young and old cells, considering that the expression level of a small set of cell identity genes was not affected by the age of the donor. It is important to keep in mind that our microscale models do not fully recapitulate the vascular niche, including tissue-specific cells and immune cells, which might contribute to changes in vascular functionality during aging and disease. The absence of these components might partially explain why we observed limited (~50%) differences in vascular functionality. Further, it is important to mention that the contribution of SMCs to the vascular barrier could be in terms of both paracrine secretion and direct support to the endothelial monolayer. While confocal images showed that iSMCs from old donors did not physically interact with iVECs, differential expression analysis highlighted that iSMCs from old donors upregulated genes which might interfere with the integrity of the barrier (e.g. *BMP2* which was identified through RNAseq and then through immunofluorescence of skin biopsies). Although we cannot separate the contribution of these two aspects, it is likely that the combination of physical support and secreted factors contributes to the observed differences in the barrier functionality in presence of young vs. old iSMCs.

Once validated in the context of physiological aging, we applied our strategy to investigate potential mechanisms involved in the vascular degeneration of HGPS, which is the leading cause of death for patients affected by this disease. HGPS is characterized by a mutation in the Lamin A/C gene causing structural defects in the nuclear envelope and leading to the appearance of signs of accelerated aging (Prokocimer et al., 2013). One of the main cardiovascular complications observed in HGPS is the loss of SMC coverage of blood vessels (Stehbens et al., 2001; Varga et al., 2006). Dysfunctional SMCs have been associated with several vascular damages, including vascular calcification (Wei et al., 2018) and hypertension (Miriyala et al., 2006). However, the molecular mechanisms leading to this vascular degeneration in HGPS and other cardiovascular diseases have not yet been completely identified.

In a recent tissue engineered blood vessel model, iPS cell-derived SMCs from a HGPS donor showed increased calcification and apoptosis when compared to cells obtained from a healthy donor (Atchison et al., 2017). However, no genetic analyses were performed to investigate potential targets or biological mechanisms involved. Herein we combined RNAseq and functional assays of vascular permeability to compare iSMCs from healthy vs. HGPS donors and we identified novel targets associated with HGPS vascular dysfunction. We did not find overlapping genes comparing old vs. young and HGPS vs. healthy cells. However, we were surprised to find that *BMP2* was upregulated in old donors while *BMP4* was highly expressed by HGPS cells. BMP2 and BMP4 share a 62% sequence similarity and are functionally equivalent. Hence, BMP2/4 dysregulation might underlie a common mechanism leading to vascular degeneration during physiological aging and disease. In this context, BMP4 is not only linked to vascular calcification. Indeed, it directly affects the stability of the endothelial barrier by increasing the vascular permeability both in vitro and in vivo (Helbing et al., 2017). Hence, BMP4 might represent a pleiotropic factor secreted by SMCs which compromises different components of blood vessels. Surprisingly, activation of BMP signaling was reported in presence of mutations of Lamin A/C and other nuclear proteins in skeletal muscle cells (Dubinska-Magiera et al., 2013). Moreover, depletion of Lamin A/C in mouse embryonic fibroblasts determined upregulation of several genes including *BMP4* (Singh et al., 2013). Together, these data suggest that Lamin A/C could be directly or indirectly involved in the expression of BMP4, hence establishing a potential link between HGPS and BMP4 dysregulation. Our measurement of BMP4 in blood serum from HGPS and age-matched healthy donors revealed opposite trends in the transition between early/late childhood. The lower level of BMP4 detected during early childhood in HGPS patients could be correlated with the unphysiological development of their musculo-skeletal system. Indeed, the largest growth rate of long bones occurs between 5 and 11 years old (Abrahamyan et al., 2008) and relies on multiple signaling molecules including BMP4. Conversely, abnormal levels of BMP4 in adulthood are correlated with vascular dysfunctions including calcification, atherosclerosis and vascular leakage. It is possible then that the higher level of BMP4 in HGPS vs. control donors during late childhood could be associated, together with other dysregulated pathways, with their cardiovascular decline. Despite these tantalizing observations, we acknowledge that additional analyses are required to give clinical significance to our findings, considering the limited number of samples that were employed (N = 7 donors per group). However, we would like to acknowledge that it is extremely difficult to obtain serum form HGPS patients due to the extremely low number of HGPS samples which are available for basic research.

Surprisingly, we also found that primary, healthy VECs upregulated *BMP4* and other genes associated with calcification, skeletal system development and extracellular matrix organization when co-cultured with HGPS fibroblasts. Although very preliminary, these data suggest that different HGPS vascular cells could autonomously upregulate or induce the expression of bone-related genes.

In addition to *BMP4*, we also found that iSMCs from HGPS donors showed upregulation of the Notch ligand *JAG1*. Notch signaling plays a critical role in vascular homeostasis and previous reports demonstrated that Jagged 1 increases the vascular permeability of the blood brain barrier (Yao et al., 2011). Furthermore, Jagged 1 was recently identified as one of the contributors of the aging signature of healthy human donors in a large study comparing protein expression and chronological age (Tanaka et al., 2018). To our knowledge, no previous studies demonstrated the dysregulation of two key mediators of vascular permeability in HGPS SMCs. Hence, our approach has the potential to identify novel therapeutic targets in a wide set of diseases characterized by cardiovascular dysfunction, including Alzheimer Disease and Diabetes. In this scenario, the use of reprogrammed vascular cells could be extended to monitor the personalized response of a patient to a given therapy. However, in order to reach these goals it will be essential to optimize current protocols promoting the full maturation of reprogrammed vascular cells and to develop high-throughput systems compatible with the functional screening of hundreds or even thousands of experimental conditions in parallel.

## Materials and methods

**Key resources table:** 

Reagent type (species) or resource	Designation	Source or reference	Identifiers	Additional information
Cell line (*Homo-Sapiens*)	Dermal fibroblasts (normal)	Coriell	Details in Supplementary file 1	https://www.coriell.org/0/Sections/Support/Global/QCcells.aspx?PgId=409
Cell line (*Homo-Sapiens*)	Dermal fibroblasts (normal and HGPS)	Progeria Research Foundation	Details inSupplementary file 1	https://www.progeriaresearch.org/wp-content/uploads/2020/04/PRF-AVAILABLE-CELL-LINES.pdf
Cell line (*Homo-Sapiens*)	Microvascular endothelial cells (normal)	Angioproteomie	Details in Supplementary file 1	https://www.angioproteomie.com/commerce/ccp1073-human-dermal-microvascular-endothelial-cells-cap-0005.htm
Cell line (*M. musculus*)	Microvascular endothelial cells (normal)	Cell Biologics	Details in Supplementary file 1	https://cellbiologics.com/index.php?route=product/product&path=2_47_89_91&product_id=2240
Transfected construct (human)	pQCXIB EGFP-MYOCD	This paper		Retroviral construct for stable expression
Transfected construct (human)	pQCXIB ETV2	This paper		Retroviral construct for stable expression
Transfected construct (human)	On Target Plus Human PALD1 siRNA (Smart Pool)	Horizon	L-026434-00-0005	
Transfected construct (human)	On Target Plus Human BMP2 siRNA (Smart Pool)	Horizon	L-011219-00-0005	
Biological sample (*Homo-sapiens*)	Human skin samples	This paper		Isolated from human donors by the Cooperative Human Tissue Network or by the Clinical and Translational Research Institute of UCSD
Antibody	Anti-human CD31 (Mouse monoclonal)	ThermoFisher	Cat# MA3100 RRID:AB_223516	IF(1:100)
Antibody	Anti-human collagen type I (Mouse monoclonal)	ThermoFisher	Cat# MA126771 RRID:AB_2081889	IF(1:250)
Antibody	Anti-human VE-cadherin (Rabbit monoclonal)	Cell Signaling	Cat#2500S RRID:AB_10839118	IF(1:100)
Antibody	Anti-human Y658 VE-cadherin (Rabbit polyclonal)	ThermoFisher	Cat#441144G RRID:AB_2533583	IF(1:100)
Antibody	Anti-human alpha smooth muscle actin (Mouse monoclonal)	eBioscience	Cat#14-9760-82 RRID:AB_2572996	IF(1:2,500)
Antibody	Anti-human calponin (Mouse monoclonal)	Santa Cruz	Cat#sc-58707 RRID:AB_781770	IF(1:100)
Antibody	Anti-human BMP2 (Rabbit polyclonal)	Novus	Cat#nBP1-19751 RRID:AB_2227877	IF(1:100)
Antibody	Anti-human Paladin (Rabbit polyclonal)	ThermoFisher	Cat#PA5-53591 RRID:AB_2645183	IF(1:100)
Antibody	Anti-human GSTM1 (Mouse Monoclonal)	ThermoFisher	Cat#MA5-17085 RRID:AB_2538556	IF(1:100)
Antibody	Anti-human BMP4 (Mouse Monoclonal)	R and D Systems	Cat#MAB3552 RRID:AB_2065677	Blocking (2 ug/ml)
Antibody	Anti-human Jagged 1 (Goat polyclonal)	R and D Systems	Cat#AF1277 RRID:AB_354713	Blocking (1 ug/ml)
Sequence-based reagent	CD31 forward	This paper	PCR primers	TGGTCAAGAAAAGCAACACAG
Sequence-based reagent	CD31 reverse	This paper	PCR primers	GATTCGCAACGGACTTCG
Sequence-based reagent	CD31 forward	This paper	PCR primers	ACAACGAGGGCATCATCAAG
Sequence-based reagent	CD31 reverse	This paper	PCR primers	GAAGTGGTAGAAAGGCTGCTG
Sequence-based reagent	BMP2 forward	This paper	PCR primers	CATGCTAGACCTGTATCGCA
Sequence-based reagent	BMP2 reverse	This paper	PCR primers	TGTTTTCCCACTCGTTTCTGG
Sequence-based reagent	BMP2 forward	This paper	PCR primers	GCCCTTCGAGCACCACGCA
Sequence-based reagent	BMP2 reverse	This paper	PCR primers	TGGCTTGTAGTGCCGCTGCTG
Commercial assay or kit	BMP4 ELISA Kit	Sigma-Aldrich	RAB0029	
Software, algorithm	Fiji			https://fiji.sc/

### Cell culture and human serum

Cell lines used in the study are reported in Supplementary file 1. Details are available in Supplementary Materials and methods. Briefly, iVECs and primary endothelial cells were cultured in EGM-2MV (Lonza) on collagen coated dishes, iSMCs were cultured in EGM-2 (Lonza) and fibroblasts were cultured in DMEM+10%FBS+1% P/S. Human primary dermal fibroblast cell lines and serum samples were obtained from The Progeria Research Foundation (PRF) Cell and Tissue Bank. The HGPS cell lines were HGADFN178, HGADFN188, HGADFN164, HGADFN143, HGADFN169, HGADFN122, HGADFN127, HGADFN367. The control cell lines were HGADFN368, GM03349, GM01652. The HGPS serum samples were 205S1, 009, 231, 224, 82, 113, 171. The control samples were 237, 148, 207, 205, 203, 197, 232. Details are reported in Supplementary file 1 and 7.

### RNAseq: library preparation and data analysis

The quality of cDNA libraries was checked with Tape Station (Agilent) and the amount of cDNA quantified through Qubit (ThermoFisher). HiSeq2500 was used to sequence the samples (single-end reading). Reads were mapped by STAR [v2.5.3a, ref: 10.1093/bioinformatics/bts635. pmid:23104886] to the hg19 reference genome with default parameters. Homer [v4.9.1, Heinz et al., 2010; http://homer.ucsd.edu/homer/] was used to quantify gene expression by counting uniquely mapped reads across all exons of RefSeq genes. Differential expression analysis was performed using edgeR for all iSMC related comparisons and DESeq2 for all iVECs related comparisons. Genes with adjusted p-value<0.05 and absolute log fold-change >1 were identified as significantly differentially expressed genes between two conditions. Gene ontology maps were generated with Metascape [http://metascape.org]. Heatmaps were generated using package gplots (https://cran.r-project.org/web/packages/gplots/index.html) in R (R Core Team (2013). R: A language and environment for statistical computing. R Foundation for Statistical Computing, Vienna, Austria. URL http://www.R-project.org/) on the row-wise z-scaled log-transformed count per million values (CPM). A pseudo-count of 5 was added to each CPM value before log-transformation to reduce noise. Dendrograms on the heatmap were calculated using Ward. D2 clustering algorithm and 1-Pearson’s correlation as distance measurement.

### Statistics

Details on number of biological replicates, independent measurements/technical replicates, data normalization and statistical tests are reported in each figure legend. Error bars are standard error of the mean (s.e.m.).

## Data Availability

Sequencing data have been deposited in GEO under accession code GSE140898. The following dataset was generated: BersiniSSchulteRHuangLTsaiHHetzerMW2019Direct reprogramming of fibroblasts identifies signatures of vascular dysfunction in physiological aging and Hutchinson-Gilford Progeria SyndromeNCBI Gene Expression OmnibusGSE140898 The following previously published dataset was used: QuertermousT2018Coronary artery disease genes SMAD3 and TCF21 promote opposing interactive genetic programs that regulate smooth muscle cell differentiation and disease riskNCBI Gene Expression OmnibusGSM317551810.1371/journal.pgen.1007681PMC619898930307970

## Additional files

Supplementary file 1. The table collects relevant information on the source of fibroblasts, SMCs and endothelial cells employed in the study.

Supplementary file 2. Details on DE genes between young vs old iVECs, young vs old iSMCs, normal vs HGPS iSMCs. Details on clustering analyses comparing iVECs, primary VECs and fibroblasts as well as iSMCs, primary SMCs and fibroblasts.

Supplementary file 3. Details on clustering analyses comparing induced vs primary cells. This dataset refers to data presented in Figure 1G and I.

Supplementary file 4. Code used for data analyses.

Supplementary file 5. Cell identity gene analysis in reprogrammed cells. The table collects relevant information on key cell identity genes expressed by reprogrammed and primary cells.

Supplementary file 6. Cell identity gene analysis in young vs old reprogrammed cells. The table collects relevant information on key cell identity genes expressed by reprogrammed cells derived from young and old donors.

Supplementary file 7. The table collects relevant information on the source of human serum employed in the study.

Transparent reporting form

## Appendix 1

### Supplementary Materials and Methods

Direct reprogramming of skin fibroblasts into vascular cells Human dermal fibroblasts documented as ‘apparently healthy individuals’ deposited from the National Institute of Aging or the NIGMS Human Genetic Cell Repository were obtained or purchased from the Coriell Institute Cell Repository (Camden, NJ, USA). HGPS patient fibroblasts were obtained from the Progeria Research Foundation (PRF). HGPS fibroblasts were characterized by a mutation in the LMNA gene, as certified by the Progeria Research Foundation (https://www.progeriaresearch.org/wp-content/uploads/2019/11/PRF-AVAILABLE-CELL-LINES-1.pdf). Both Coriell and PRF certify that their cells are mycoplasma free at time of purchase. RNA-seq analysis further confirmed the cell identity of these cells. Details are available in Supplementary file 1 and in the Key Resources Table. Fibroblasts cell lines were also derived from human patients who gave written informed consent and consent to publish as outlined in the ethical approval IRB 15–0004, which was approved by the Salk Institute IRB board. Biopsies were obtained from the upper arm of patients by the UCSD Clinical and Translational Research Institute. Biopsies were dissected and placed in growth medium containing collagenase for 1 h at 37C. Samples were resuspended in growth medium and distributed to gelatin-coated plates. Fibroblast cultures began to grow out from tissue approximately 1 week after seeding. Cell identity was confirmed by RNA-seq. Fibroblasts were infected with retroviral virus to express *EGFP-MYOCD* (iSMCs) or *ETV2* (iVECs). Infected cells were selected by blasticidin treatment 2 days after infection. iSMCs were kept in culture with blasticidin for an additional 7 days before analysis, while iVECs were cultured with blasticidin for additional 14 days before sorting for CD31 positive expression (BD Influx System (BD Biosciences)). iVECs were then kept in culture for additional 5 days before analysis. In vitro 3D model of microvascular networks 3D models were developed based on previous studies by our group (Bersini et al., 2016). According to the specific experiment, 12 Mcells/ml iVECs or primary VECs and 12 Mcells/ml iSMCs or primary fibroblasts were suspended in EGM-2MV + thrombin (4 U/ml) at a 1:1 ratio. The cell suspension was 1:1 mixed with a 5 mg/ml solution of human fibrinogen. 30 µl of the resulting mix were pipetted into ibidi slides and incubated with EGM-2MV. The final cell density was equal to 3 Mcells/ml for each cell population. Cell cultures were kept in humidified incubators (37C, 5% CO2) for 14 days and monitored daily through epifluorescence and brightfield microscopy. Medium was replaced every 2 days avoiding any contact with the 3D fibrin scaffold. Immunofluorescence Samples were washed with warm PBS followed by 10 min fixation with 3% PFA. After washing with PBS, samples were incubated with 0.1% Triton-X 100 for 10 min. Following PBS washing, samples were incubated with 5% bovine serum albumin (BSA) at room temperature (RT) for 2 h, followed by overnight incubation at 4C with primary antibody diluted in 5% BSA solution. Samples were washed three times in PBS for 10 min each and then incubated with secondary antibody diluted in PBS for 3 h at RT. Finally, samples were washed three times in PBS for 10 min each. The following antibodies and dilutions were used: 1:100 anti-human CD31, MA3100, mouse monoclonal, ThermoFisher; 1:250 anti-human collagen type I, MA126771, mouse monoclonal, ThermoFisher; 1:100 anti-human vascular endothelial (VE)-cadherin, 2500S, rabbit monoclonal, Cell Signaling; 1:100 anti-human Y658 VE-cadherin, 441144G, rabbit polyclonal, ThermoFisher; 1:2500 anti-human alpha-smooth muscle actin (α-SMA), 14-9760-82, mouse monoclonal, eBioscience; 1:100 anti-human calponin 1, sc-58707, mouse monoclonal, SantaCruz; 1:100 anti-human BMP2, nBP1-19751, rabbit polyclonal, Novus. Secondary antibody used: 1:200 alexafluor 647 (H+L IgG) goat anti-mouse or donkey anti-rabbit. Nuclei were counterstained with 1:500 Hoechst while 1:200 555-Phalloidin (Cytoskeleton) was used to stain actin. Samples were imaged with Leica SP8 laser scanning confocal microscope. Immunofluorescence of human skin samples Human skin samples were obtained from two different sources to take into account variability. Cooperative Human Tissue Network (CHTN) was contacted after IRB approval to obtain samples from deceased donors (max 24 h after death). Samples were collected from a young donor (45 years old) and an old donor (68 years old). In addition, skin biopsies were performed at the Clinical and Translational Research Institute of UCSD upon IRB approval (Protocol #15–0004). Samples were obtained from a 21 and a 72 years old donor. Samples were kept free-floating in formalin at 4C for 24 h, then washed in PBS at 4C for 10 min under rotation and finally incubated in 30% sucrose at 4C for at least 24 h for cryopreservation. Samples were then embedded in OCT and stored at −80°C until further processing. 20 µm thick tissue slices were cut by trained personnel at the UCSD Histology Core. For staining, slices were kept at RT for 10 min, washed twice with PBS for 5 min and then incubated with blocking solution (1% BSA, 1% skin fish gelatin, 0.3% Triton-X 100) at RT for 1 h. Primary antibody was then incubated at RT overnight. Samples were washed three times in PBS for 5 min each and then incubated with secondary antibody at RT for 1 h. Finally, samples were washed three times with PBS for 5 min each. A drop of mounting medium for immunofluorescence (Vectashield, H-1000) was put on each sample, which was then covered with a 1.5# thickness coverslip. Nail polish was used to seal each coverslip to prevent evaporation. The following primary antibodies were used: 1:100 anti-human CD31, MA3100, mouse monoclonal, ThermoFisher; 1:250 anti-human alpha-smooth muscle actin (α-SMA), 14-9760-82, mouse monoclonal, eBioscience; 1:100anti-human BMP2, nBP1-19751, rabbit polyclonal, Novus; 1:100 anti-human Paladin 1, PA5-53591, rabbit polyclonal, ThermoFisher;1:100 anti-human GSTM1, MA5-17085, mouse monoclonal, ThermoFisher. The following secondary antibodies were used: 1:200 alexafluor 647 (H+L IgG) goat anti-mouse or donkey anti-rabbit; 1:200 alexafluor 568 (H+L IgG) goat anti-mouse or goat anti-rabbit. Nuclei were counterstained with 1:500 Hoechst. Samples were imaged the same day using a Leica SP8 laser scanning confocal microscope, setting the same configuration (laser power, digital gain, digital offset) for each condition requiring a direct comparison. Image acquisition, analysis and quantification Images were analyzed using the Fiji software (https://fiji.sc/). For skin biopsies, it is important to highlight that skin capillaries do not grow along a preferential direction (as for example in the skeletal muscle along muscle fibers). Hence, we found both cross-sections of capillaries and longitudinally-sectioned vessels. Quantification of immunofluorescence images was performed by calculating the average fluorescence intensity of each selected antibody co-localized with CD31 signal for iVECs or αSMA for iSMCs in multiple sections. The average intensity of PALD1 or BMP2 in the young samples was then calculated and normalized to 100%. All images were acquired with the same laser configuration from tissue sections characterized by the same thickness and obtained from the same anatomical location. Quantification of the BMP2 signal in co-culture experiments of iSMCs reprogrammed from young vs. old donors was performed by measuring the average fluorescence intensity for individual cells (young vs. old cells were discriminated by using the MYOCD fluorescence signal (EGFP or mCherry in this specific experiment)). Note that the MYOCD fluorescence signal (EGFP or mCherry) was then exchanged between young vs. old cells to ensure that the construct was not affecting the expression of BMP2 and the obtained results are an average of both conditions. Regarding Y658 VE-cadherin, the area fraction was quantified by thresholding the fluorescence signal and then normalizing by the number of nuclei per each region of interest (ROI). Microfluidic experiments and vascular permeability quantification Microfluidic devices were designed based on modifications of previous models (Jeon et al., 2014). The design is based on a central channel embedding a cell-laden matrix (e.g. the matrix contains iSMCs or is acellular in case of experiments only considering iVECs) and two lateral channels filled with EGM-2MV media. Each channel is 200 µm thick. The Smooth-Cast 310 resin was used to create master molds. Microfluidic devices were then fabricated with poly-dimethyl-siloxane (PDMS) and sterilized in autoclave. Devices were bonded to #1 thickness sterile coverslips using an oxygen plasma cleaner. The central channel was filled with 7.5 mg/ml fibrin matrix (acellular or embedding 1.5 Mcells/ml iSMCs). The day after matrix seeding, 5 Mcells/ml iVECs were flowed through one of the lateral channels to create a monolayer covering the interface with the central channel. After 3 additional days, a monolayer of iVECs covered 100% of the interfaces between the lateral channel and the central channel. Devices were then used for vascular permeability quantification or fixed for subsequent immunofluorescence staining. For vascular permeability quantification, microfluidic devices were only used after formation of a complete endothelial monolayer covering the interface with the central channel. For live imaging, the medium was aspirated from the channels and the devices were put on the stage of a Leica SP8 laser scanning confocal microscope. Next, 10 µl 70 kDa fluorescent dextran were injected in the vascularized lateral channel and multiple stacks were acquired in different locations of each device every 3 min. For quantification of vascular permeability, we used a previously established protocol (Jeon et al., 2015) whereby the change of fluorescent intensity in a 50 µm x 200 µm area across the vascular interface between lateral and central channel was quantified. The following equation was considered:

$$P=\frac{1}{I_{i}-I_{b}}\left(\frac{I_{f}-I_{i}}{\Delta t}\right)\times w$$

where *P* is vascular permeability, *I_f_* and *I_i_* are the intensity of the signal at the final and initial time point, *I_b_* is the background intensity, *Δt* is the time interval between final and initial time point and *w* is the width of the vascularized channel. The minimum 3 min interval between consecutive acquisition is necessary to avoid photobleaching. The same experimental setup was used in experiments embedding cells silenced for *PALD1* (VECs) or *BMP2* (SMCs). Cells were silenced for *PALD1* or *BMP2* using a pool of 4 siRNA for each target (Dharmacon). The final siRNA concentration was 100 nM. siLentFect (Biorad) was used as lipid reagent for transfection. Cells were embedded in microfluidic chamber 48 to 72 h after transfection and upon validation of KD efficiency through Western Blot or qPCR. The same experimental setup was also used in experiments with blocking antibodies with iSMCs from HGPS patients embedded in the 3D matrix and VECs forming a monolayer in the adjacent channel. Blocking antibodies targeting BMP4 (mouse monoclonal IgG1, MAB3552, R and D Systems) or Jagged 1 (goat polyclonal IgG, AF1277, R and D Systems) were introduced in the system for specific experiments. The antibody treatment started 1 day after seeding iSMCs and continued throughout the entire duration of the experiment. BMP4 blocking antibody was diluted in EGM-2MV at a final concentration of 2 µg/ml, while the Jagged 1 antibody final concentration was 1 µg/ml. Media with antibodies were replaced every day. qPCR The following primers were used:

*CD31* forward: TGGTCAAGAAAAGCAACACAG
*CD31* reverse: GATTCGCAACGGACTTCG
*CDH5* forward: ACAACGAGGGCATCATCAAG
*CDH5* reverse: GAAGTGGTAGAAAGGCTGCTG
*BMP2* forward: CATGCTAGACCTGTATCGCA
*BMP2* **r**everse: TGTTTTCCCACTCGTTTCTGG
*RPL4* forward: GCCCTTCGAGCACCACGCA
*RPL4* reverse: TGGCTTGTAGTGCCGCTGCTG

ELISA The human BMP4 ELISA kit (Sigma-Aldrich, RAB0029) was used to measure the level of circulating protein in blood serum from HGPS and age-matched healthy human donors. The same kit was used to measure the level of circulating protein in blood serum from HGPS, age-matched healthy and old healthy mice.
