# Supplementary material for: Direct reprogramming of human smooth muscle and vascular endothelial cells reveals defects associated with aging and Hutchinson-Gilford progeria syndrome
Source: eLife. 2020 Sep 8;9:e54383. doi: 10.7554/eLife.54383 (PMC7478891; doi:10.7554/eLife.54383)
Supplement: Supplementary file 1. [file elife-54383-supp1.docx]

**Supplementary File 1**. The table collects relevant information on the source of fibroblasts, SMCs and endothelial cells employed in the study.

| **Code** | **Cell type** | **Age** | **Source** | **Race** | **Sex** | **Apparently healthy** |
| --- | --- | --- | --- | --- | --- | --- |
| 22CTRI-APR12,16 (lab derived) | Fibroblast | Young, 22 y.o. | Skin, arm | Caucasian | Male | Yes |
| 23CTRI-APR12;16  (lab derived) | Fibroblast | Young, 23 y.o. | Skin, arm | Middle Eastern | Male | Yes |
| 25CTRI-APR12;16  (lab derived) | Fibroblast | Young, 25 y.o. | Skin, arm | Asian | Female | Yes |
| 62CTRI-APR1216  (lab derived) | Fibroblast | Old, 62 y.o. | Skin, arm | Caucasian | Male | Yes |
| 64CTRI-APR1216  (lab derived) | Fibroblast | Old, 64 y.o. | Skin, arm | Caucasian | Male | Yes |
| 67CTRI-ARP1916  (lab derived) | Fibroblast | Old, 67 y.o. | Skin, arm | Caucasian | Female | Yes |
| GM03523 (Coriell) | Fibroblast | Young, 21 y.o. | Skin, neck | Caucasian | Male | Yes |
| GM23815 (Coriell) | Fibroblast | Young, 22 y.o. | Skin | Caucasian | Male | Yes |
| GM03651 (Coriell) | Fibroblast | Young, 25 y.o. | Skin, arm | Caucasian | Female | Yes |
| GM02674 (Coriell) | Fibroblast | Young, 29 y.o. | Skin | Caucasian | Female | Yes |
| GM23973 (Coriell) | Fibroblast | Young, 19 y.o. | Skin | Caucasian | Male | Yes |
| AG09605 (Coriell) | Fibroblast | Young, 30 y.o. | Skin, arm | Caucasian | Male | Yes |
| GM03529 (Coriell) | Fibroblast | Old, 66 y.o. | Skin, thigh | Black | Male | Yes |
| GM01681 (Coriell) | Fibroblast | Old, 70 y.o. | Skin, arm | Caucasian | Male | Yes |
| GM09918 | Fibroblast | Old, 78 y.o. | Skin | Caucasian | Male | Yes |
| AG16102 (Coriell) | Fibroblast | Old, 69 y.o. | Skin, arm | Caucasian | Male | Yes |
| GM03524 (Coriell) | Fibroblast | Old, 67 y.o. | Skin, leg | Black | Female | Yes |
| AG11017 (Coriell) | Fibroblast | Old, 87 y.o. | Skin, arm | Black | Male | Yes |
| AG13065 (Coriell) | Fibroblast | Old, 64 y.o. | Skin, arm | Caucasian | Male | Yes |
| HGADFN178 (Progeria Research Foundation (PRF)) | Fibroblast | HGPS, 6 y.o. | Skin |  | Female | HGPS |
| HGADFN188 (PRF) | Fibroblast | HGPS, 2 y.o. | Skin |  | Female | HGPS |
| HGADFN164 (PRF) | Fibroblast | HGPS, 4 y.o. | Skin |  | Female | HGPS |
| HGADFN143 (PRF) | Fibroblast | HGPS, 8 y.o. | Skin |  | Male | HGPS |
| HGADFN169 (PRF) | Fibroblast | HGPS, 8 y.o. | Skin |  | Male | HGPS |
| HGADFN122 (PRF) | Fibroblast | HGPS, 5 y.o. | Skin |  | Female | HGPS |
| HGADFN127 (PRF) | Fibroblast | HGPS, 3 y.o. | Skin |  | Female | HGPS |
| HGADFN367 (PRF) | Fibroblast | HGPS, 3 y.o. | Skin |  | Female | HGPS |
| HGADFN368 (PRF) | Fibroblast | Ctrl, 31 y.o. | Skin |  | Female | Yes |
| GM03349 (PRF) | Fibroblast | Ctrl, 10 y.o. | Skin |  | Male | Yes |
| GM01652 (PRF) | Fibroblast | Ctrl, 11 y.o. | Skin |  | Female | Yes |
| SRX4161710 (GEO source) | Smooth muscle cell |  | Coronary artery |  |  | Yes |
| SRX4161711 (GEO source) | Smooth muscle cell |  | Coronary artery |  |  | Yes |
| SRX4161712 (GEO source) | Smooth muscle cell |  | Coronary artery |  |  | Yes |
| cAP0005GFP (Angioproteomie) | Microvascular endothelial cell |  | Skin |  |  | Yes |
| A57-6064  (CellBiologics) | Mouse endothelial cell | 78 weeks old | Skin |  |  | Yes |
| A57-6023  (CellBiologics) | Mouse endothelial cell | 78 weeks old | Brain |  |  | Yes |
| A57-6221  (CellBiologics) | Mouse endothelial cell | 78 weeks old | Bone marrow |  |  | Yes |
| C57-6064  (CellBiologics) | Mouse endothelial cell | 8 weeks old | Skin |  |  | Yes |
| C57-6064  (CellBiologics) | Mouse endothelial cell | 8 weeks old | Brain |  |  | Yes |
| C57-6064  (CellBiologics) | Mouse endothelial cell | 8 weeks old | Bone marrow |  |  | Yes |
