# Supplementary material for: Direct reprogramming of human smooth muscle and vascular endothelial cells reveals defects associated with aging and Hutchinson-Gilford progeria syndrome
Source: eLife. 2020 Sep 8;9:e54383. doi: 10.7554/eLife.54383 (PMC7478891; doi:10.7554/eLife.54383)
Supplement: Supplementary file 3. — This dataset refers to data presented in Figure 1G and I. [file elife-54383-supp3.docx]

**Supplementary File 3**. The table collects relevant information on key cell identity genes expressed by reprogrammed cells derived from young and old donors.

| **Gene** | **Young vs. old iVECs [log2FC]** | **[FDR]** |
| --- | --- | --- |
| *FLT1* | -0.478 | 0.999 |
| *KDR* | 0.160 | 0.999 |
| *ROBO4* | 0.052 | 0.999 |
| *CDH5* | -0.408 | 0.999 |
| *NECTIN3* | -0.267 | 0.999 |
| *TJP2* | -0.030 | 0.999 |

| **Gene** | **Young vs. old iSM cells [log2FC]** | **[FDR]** |
| --- | --- | --- |
| *ACTA2* | -0.568 | 0.999 |
| *TAGLN* | -0.102 | 0.999 |
| *CNN1* | 0.124 | 0.999 |
| *MYLK* | -0.070 | 0.999 |
