# Supplementary material for: Direct reprogramming of human smooth muscle and vascular endothelial cells reveals defects associated with aging and Hutchinson-Gilford progeria syndrome
Source: eLife. 2020 Sep 8;9:e54383. doi: 10.7554/eLife.54383 (PMC7478891; doi:10.7554/eLife.54383)
Supplement: Supplementary file 4. [file elife-54383-supp4.docx]

**Supplementary File 4**. The table collects relevant information on the source of human serum employed in the study.

| **Code** | **Age** | **Sex** | **Diagnosis** |
| --- | --- | --- | --- |
| 205 S1 | 5 | Female | HGPS |
| 237 | 5.1 | Male | Ctrl |
| 009 | 8.5 | Male | HGPS |
| 148 | 8.8 | Female | Ctrl |
| 231 | 8.6 | Female | HGPS |
| 207 | 9 | Female | Ctrl |
| 224 | 9.9 | Male | HGPS |
| 205 | 10.4 | Female | Ctrl |
| 82 | 11.8 | Male | HGPS |
| 203 | 11.8 | Male | Ctrl |
| 113 | 12.2 | Female | HGPS |
| 197 | 12.1 | Female | Ctrl |
| 171 | 17.7 | Male | HGPS |
| 232 | 17.7 | Female | Ctrl |
