# Supplementary material for: Direct reprogramming of human smooth muscle and vascular endothelial cells reveals defects associated with aging and Hutchinson-Gilford progeria syndrome
Source: eLife. 2020 Sep 8;9:e54383. doi: 10.7554/eLife.54383 (PMC7478891; doi:10.7554/eLife.54383)
Supplement: Supplementary file 5. — The table collects relevant information on key cell identity genes expressed by reprogrammed and primary cells. [file elife-54383-supp5.docx]

**Supplementary File 5**. The table collects relevant information on key cell identity genes expressed by reprogrammed and primary cells.

| **Gene** | **Primary VECs vs. Fibroblasts [log2FC]** | **iVECs vs. Fibroblasts [log2FC]** |
| --- | --- | --- |
| *FLT1* | 2.5 | 4.87 |
| *TIE2* | 4.69 | 1.86 |
| *KDR* | 6.62 | 6.38 |
| *CD31* | 6.85 | -0.5 |
| *ROBO4* | 6.61 | 3.92 |
| *CDH5* | 6.7 | 4 |
| *NECTIN3* | -0.36 | 2 |
| *TJP1* | 1.07 | -0.43 |
| *SLUG* | -1.1 | -1.24 |
| *TWIST2* | -1.24 | -2.03 |
| *TWIST1* | -1.43 | -1.54 |
| *FSP1* | -1.58 | -1.55 |

| **Gene** | **Primary SMCs vs. Fibroblasts [log2FC]** | **iSMCs vs. Fibroblasts [log2FC]** |
| --- | --- | --- |
| *ACTA2* | 2.04 | 4.95404236 |
| *TAGLN* | 0.54 | 1.941497224 |
| *MYH10* | 1.34 | 0.456915118 |
| *FSP1* | -6.21 | -3.829971681 |
