# Supplementary material for: Direct reprogramming of human smooth muscle and vascular endothelial cells reveals defects associated with aging and Hutchinson-Gilford progeria syndrome
Source: eLife. 2020 Sep 8;9:e54383. doi: 10.7554/eLife.54383 (PMC7478891; doi:10.7554/eLife.54383)
Supplement: Peer review history

# Decision letter

**DOI:** 10.7554/eLife.54383.sa1

**Affiliations:** UT Health San AntonioUnited States; Washington University School of MedicineUnited States

In the interests of transparency, eLife publishes the most substantive revision requests and the accompanying author responses.

Thank you for choosing to send your work, "Direct reprogramming human vascular cells reveals defects associated with aging and Hutchinson-Gilford Progeria Syndrome", for consideration at *eLife*. Your article has been reviewed by three peer reviewers, one of whom is a member of our Board of Reviewing Editors, and the evaluation has been overseen by Jessica Tyler as the Senior Editor.

Bersini et al. provide a conceptual advance in the use of direct reprogramming to model age-associated dysfunction of vascular cells. This is a novel and exciting proposed approach to study mechanisms of age-related vascular dysfunction and may provide actionable in vitro models of age-related vascular disease. The data presented, however, do not support efficacy of the reprogramming approach at the transcriptional or functional level. Critical validation of the approach is lacking. In the functional comparisons between reprogrammed cells from young and old donors, it is unclear that reprogramming efficiencies are comparable. Substantial weaknesses in the statistical analyses of the data were identified. Some of the supplementary materials provided may be superfluous and could be replaced by informative panels included in the main figures. BMP2 is identified in one set of experiments, but subsequent experiments focus on a different gene, BMP4. From an RNA-seq experiment that was performed data from only 2 genes are shown. Overall, while the focus of the studies reported is of high interest, these and other weaknesses decrease the priority for publication of these studies in their current form. A revised manuscript adequately addressing the main concerns raised in the peer-review process, that will require additional experimental work, might be suitable for publication. A revised Tools and Resources article also needs to provide additional insights into how the authors' reprogramming techniques and biological resources will advance the field.

Reviewer #1:

Bersini et al. report the generation of induced vascular endothelial cells and smooth muscle cells by direct reprogramming of skin fibroblasts through enforced expression of single master regulator genes for the endothelial and smooth muscle fates respectively. The stated goal of the study is to characterize and validate directly reprogrammed vascular cells, then use to ascertain age-specific differences in their biology. The goal is important since the authors state that transcriptomic studies on direct reprogrammed cells are lacking. The studies in directly reprogrammed skin fibroblasts hinge on the effectiveness of direct reprogramming. This is not supported by the transcriptomic data of Figure 1G and I. Even though (as the author state) induced cells do not express all cell identity markers of the primary cell types that they model, the paucity of cell type-specific genes that are expressed in the induced smooth muscle and vascular endothelial cells that are presented in Figure 1G strongly suggest that these cells may still be largely of fibroblast nature. It could be argued that a handful of vascular-specific genes are sufficient to direct the functionally relevant reprogramming of fibroblasts. However, the lack of direct comparisons in functional barrier assays (i.e. lack of data from “negative” and “positive” control samples using primary fibroblasts (negative control) and primary vascular endothelial or smooth muscle cells (positive controls)) in all subsequent studies that use cells directly reprogrammed from young and old donors make interpretation of those data difficult. Further, the differences in permeability between barriers established with induced cells from old and young donors are very small, and may be related to small differences in integrity or health of induced cells that retain (as suggested by the transcriptomic studies) most of their fibroblast-like features. These are important concerns that need to be addressed.

Reviewer #2:

The current manuscript by Bersini et al. provides a conceptual advance in the utility of direct reprogramming and modeling of aging-associated dysfunction in SMCs and VECs. Interestingly, the authors identified *BMP2* and *PALD1* as genes differentially regulated in aged iSMC and iVEC, respectively, which remarkably was reflected in human biopsy samples. By taking a similar experimental approach, the authors define the upregulated expression of BMP4 in reprogrammed cells from Hutchinson-Gilford Progeria Syndrome (HGPS) patients, which underlies dysfunction of iSMCs from HGPS patients. The idea underlying the study itself and the perspective of utilizing iSMCs/iVECs for studying the effect of aging is exciting. The manuscript in its current form, however, will benefit from clarifications for some of the data presented, which would further strengthen the author's conclusions.

Based on the heatmap data shown in Figure 1, it is not clear whether the transcriptome of reprogrammed cells represent fully reprogrammed and functional iVECs/iSMCs. The expression data indicate that both iSMCs and iVECs express more genes in common with fibroblasts than with primary VECs or SMCs. The authors state "transition towards full mature cells." The readers will be better informed if the authors clarify what is the measurement for transcriptomics and function for partial versus fully reprogrammed cells, or present additional data that either reject or support fully mature versus partial reprogramming. It is understandable that the goal here is to induce SMCs or VECs to the point that can capture some of the age-associated changes, not necessarily generating cells that will be identical to primary cells. Still, this will be useful information to the readers.

The choice of some of the SMC markers was not clear. For example, Calponin (shown in Figure 1B) appears to be expressed in cells that do not express the reprogramming factor, MYOCD, suggesting that Calponin could be also expressed in human fibroblasts. As such, it will be informative to the readers if the authors show the same immunostaining data for Calponin in fibroblasts.

The main focus in Figure 2 is to define age-associated changes modeled through direct reprogramming of fibroblasts of young and old age groups. It is then essential to provide experimental evidence that the comparative analyses were performed in the realm of similar reprogramming efficiencies between young and old groups. This point is particularly crucial as fibroblasts from different ages may not reprogram with the same efficiency.

It will be good to clarify whether the difference in dextran permeability between young and old cells shown in Figure 2H may be influenced by differences in reprogramming efficiency. Looking at Figure 2H, there appears to be a difference in dextran permeability, but the raw data in Figure S6 show there are very few SMA+ cells (just one red cell) in the old sample and none at the interface with iVECs, in contrast to the young sample. To further dissect the difference in permeability due to aging, not the lack of reprogrammed cells in the old samples, it will be informative to provide clear pictures showing the reprogrammed cells at the interface of the permeability assay.

As the end of Figure 2 was currently left with the correlation studies, additional mechanistic insights into the identified DE genes will greatly signify the authors' findings, for example by either i) reducing the expression of *BMP2* or *PALD1* in old cells, or ii) overexpressing those aging-associated genes in young cells to pinpoint whether the identified DE genes are underlying the observed phenotypes.

BMP2 was discovered to be upregulated in aging in Figure 2, but all the HGPS experiments in Figure 3 were associated with a different gene, BMP4. Based on the existing datasets, is BMP4 also upregulated in aged induced cells? Also, JAG1 is introduced in Figure 3, while there is no mention of *PALD1* from Figure 2. The text will benefit from a more in-depth discussion of the link between findings of Figure 2 to Figure 3.

Reviewer #3:

Direct reprogramming of fibroblasts from young, aged, and HGPS donors into vascular endothelial cells (VECs) and smooth muscle cells (SMCs), combined with transcriptomics analyses and co-culture assays, is a novel and interesting approach to study age-related alterations in EC-SMC crosstalk and discover new potential therapeutic targets to fight vascular dysfunction in normal and premature aging. However, RNAseq results from the physiological aging-related part lacks functional validation, and RNAseq results from the HGPS-related part presents weak in vivo/ex vivo validation.

1) Regarding cells derived from normally aged donors, the authors show validation of three hits obtained from the RNAseq assays by immunofluorescence analysis of skin biopsies. However, no studies were performed to analyze the functional significance of such expression changes on endothelial permeability, as done for HGPS-related studies (e.g., using blocking antibodies or shRNA).

2) Although the HGPS-related part of the paper includes in vitro functional studies, ex vivo and in vivo validation of the RNAseq results appears rather limited. BMP4 ELISA data in Figure 2B comparing age-matched controls and HGPS patients is very far from reaching statistical significance (p=0.4), thus making very difficult to draw conclusion. I concur with the authors that these experiments are very challenging considering the limited number of available HGPS samples, and mouse studies may help overcome this limitation. Regarding mouse studies in Figure 3C, the authors used the t-test to compare serum BMP4 levels in young, aged and HGPS groups. However, t-test is not appropriate for studies including more than 2 experimental groups. These results should be re-analyzed using ANOVA and post-hoc analysis. Given the limitation of the human studies, a more thorough validation using the mouse model would significantly contribute to strengthen the biological significance of the results. For example, it would be very informative to perform qPCR and/or immunofluorescence assays to assess BMP4 and Jagged 1 levels in mouse arteries. I also miss experiments to examine in vivo endothelial permeability and the effects of neutralizing anti-BMP4 and anti-Jagged 1 antibodies in the mouse models of normal and premature aging.

3) Progerin expression should confirmed in HGPS-derived cells.

4) Several references appear to be incorrect in the introductory paragraph in the section of Results focused on HGPS. The authors state: "In particular, HGPS is characterized by dysfunctional SMCs, which promote vascular calcification (Wei et al., 2018), hypertension (Miriyala et al., 2006) and loss of vascular barrier functionality (Helbing et al., 2017)". The cited papers address the role of BMPs in vascular calcification, hypertension and loss of vascular barrier functionality, but I believe none of them include studies with either HGPS patients or HGPS experimental models. The same comment regarding the studies by Wei et al., 2018 and Miriyala et al., 2006 applies to the Discussion: "The main cardiovascular complications observed in HGPS patients include loss of SMC coverage of blood vessels (Varga, et al., 2006), abnormal vascular calcification (Wei, et al., 2018) and hypertension (Miriyala, et al., 2006)." Also note that all results in Varga et al., 2006 were derived from a mouse model of HGPS, not from HGPS patients, therefore references for SMC loss in patients should be cited instead of Varga et al., 2006 (or 1-2 review articles).

5) Figure 1A, B: Quantitative data should be presented (eg. % aSMA/Myocardin-immunoreactive and % Calponin/Myocardin-immunoreactive cells).

6) Figure 1D: The authors state that iSMCs contribute to the formation of microvascular networks in 3D fibrin matrices (Figure 1D), but no control without iSMCs is shown, which is needed to ascertain if iSMC promote this process.

7) The images in Figure 2E and 2F do not seem to be very representative according to the quantitative data shown in Figure 2C and 2D (massive upregulation in images, but only 2-fold induction in the graphs).

8) Figure 2G-J: To confirm that age-related increased permeability is due to compromised VEC junctions, confocal microscopy experiments should be performed in young and old VECs to visualize junctions within neighbouring cells, ideally in monoculture and in co-culture with young and old SMCs.

9) Figure 3D, F and G: The experiments include 3 groups, therefore t-test is not adequate. Results should be re-analyzed using ANOVA and post-hoc analysis.

10) Figure 3H-J: Quantitative results should be shown.

11) Figure 2—figure supplement 3: Quantitative results of *GSTM1* expression in young versus old ECs should be shown (as done for *BMNP2* and *PALD1* in Figure 2C and 2D, respectively.

[Editors’ note: further revisions were suggested prior to acceptance, as described below.]

Thank you for submitting your article "Direct reprogramming human vascular cells reveals defects associated with aging and Hutchinson-Gilford Progeria Syndrome" for consideration by *eLife*. Your article has been reviewed by three peer reviewers, one of whom is a member of our Board of Reviewing Editors, and the evaluation has been overseen by Jessica Tyler as the Senior Editor.

The reviewers have discussed the reviews with one another and the Reviewing Editor has drafted this decision to help you prepare a revised submission.

As the editors have judged that your manuscript is of interest, but as described below that additional experiments are required before it is published, we would like to draw your attention to changes in our revision policy that we have made in response to COVID-19 (https://elifesciences.org/articles/57162). First, because many researchers have temporarily lost access to the labs, we will give authors as much time as they need to submit revised manuscripts. We are also offering, if you choose, to post the manuscript to bioRxiv (if it is not already there) along with this decision letter and a formal designation that the manuscript is "in revision at *eLife*". Please let us know if you would like to pursue this option. (If your work is more suitable for medRxiv, you will need to post the preprint yourself, as the mechanisms for us to do so are still in development.)

Summary:

Direct reprogramming of skin fibroblasts is a valuable strategy to obtain large amounts of specific cell types that are centrally involved in human disease processes. This manuscript reports direct reprogramming of skin fibroblasts to address age-specific and disease-specific changes in vascular cells during normative aging and in accelerated aging in Hutchinson-Gilford Progeria Syndrome. The authors report the transcriptional and functional characterization of vascular cell types generated by direct reprogramming. This strategy may have substantial impact by providing a tool to study changes in vascular function and potentially that of other tissues during physiological and pathological aging.

Essential revision requirements:

The authors addressed most comments appropriately with new experimental results or new statistical analyses. However, the question of similar reprogramming efficiency between young and old samples (an essential point to the manuscript), was still not fully answered. In the revised manuscript, the authors compared the expression of ETV2 and MYOCD and found no statistical difference. However, these are comparisons of the expression level of reprogramming factors, not the reflection of similar reprogramming efficiency between young and old samples – that is, whether the reprogrammed state into iVECs or iSMC as an output, is similar between young and old samples. This is a central point that needs to be addressed.

# Reply: Author response

**DOI:** 10.7554/eLife.54383.sa2

[Editors’ note: The authors appealed the original decision. What follows is the authors’ response to the first round of review.]

Bersini et al. provide a conceptual advance in the use of direct reprogramming to model age-associated dysfunction of vascular cells. This is a novel and exciting proposed approach to study mechanisms of age-related vascular dysfunction and may provide actionable in vitro models of age-related vascular disease. The data presented, however, does not support efficacy of the reprogramming approach at the transcriptional or functional level. Critical validation of the approach is lacking. In the functional comparisons between reprogrammed cells from young and old donors, it is unclear that reprogramming efficiencies are comparable. Substantial weaknesses in the statistical analyses of the data were identified. Some of the supplementary materials provided may be superfluous and could be replaced by informative panels included in the main figures. BMP2 is identified in one set of experiments, but subsequent experiments focus on a different gene, BMP4. From an RNA-seq experiment that was performed data from only 2 genes are shown. Overall, while the focus of the studies reported is of high interest, these and other weaknesses decrease the priority for publication of these studies in their current form. A revised manuscript adequately addressing the main concerns raised in the peer-review process, that will require additional experimental work, might be suitable for publication. A revised Tools and Resources article also needs to provide additional insights into how the authors' reprogramming techniques and biological resources will advance the field.

We have extensively revised the previous version of the manuscript by performing additional analyses and experiments. Specifically, we have addressed the concerns regarding the reprogramming efficiency of cells derived from young and old donors and the differentiation level of directly reprogrammed cells. We have performed new statistical analyses suggested by reviewers #2 and #3 and we have provided additional quantifications such as pathway analysis for the RNAseq data on organ-specific mouse VECs, expression of *GSTM1* in skin biopsies, expression of Calponin in reprogrammed SMCs and analysis of endothelial cell-cell junction quality.

We have also addressed comments related to the validation of the approach: i) inclusion of negative (fibroblasts) and positive (primary VECs) controls for the vascular barrier assay; ii) permeability measurements with *PALD1* and *BMP2* knock-down on primary cells (both VECs and SMCs) to show the functional relevance of these genes.

We also provide a clear rationale for the transition from physiological aging to HGPS (including a discussion on the shift from *BMP2* to *BMP4*) and a discussion on the contribution of the reprogramming approach for the advancement of the field.

Finally, in a specific comment by reviewer #3 regarding the need for additional statistical analyses for the level of BMP4 in mouse serum, the reviewer suggested that we perform in vivo experiments to test vascular permeability after treatment with blocking antibody. We agree with the reviewer that an in vivo validation could pave the way for the identification of a robust biomarker for vascular degeneration in HGPS patients (with potential consequences for the development of new therapies). Unfortunately, we only have access to the serum of this mouse model, not the mice themselves. We also feel that these experiments might go beyond the scope of this manuscript as a *Tool and Resource* article, which is “assessed in terms of the potential to facilitate experiments that address problems that to date have been challenging or even intractable”, as reported in the guide for authors.

Reviewer #1:

Bersini et al. report the generation of induced vascular endothelial cells and smooth muscle cells by direct reprogramming of skin fibroblasts through enforced expression of single master regulator genes for the endothelial and smooth muscle fates respectively. The stated goal of the study is to characterize and validate directly reprogrammed vascular cells, then use to ascertain age-specific differences in their biology. The goal is important since the authors state that transcriptomic studies on direct reprogrammed cells are lacking. The studies in directly reprogrammed skin fibroblasts hinge on the effectiveness of direct reprogramming. This is not supported by the transcriptomic data of Figure 1G and I. Even though (as the author state) induced cells do not express all cell identity markers of the primary cell types that they model, the paucity of cell type-specific genes that are expressed in the induced smooth muscle and vascular endothelial cells that are presented in Figure 1G strongly suggest that these cells may still be largely of fibroblast nature. It could be argued that a handful of vascular-specific genes are sufficient to direct the functionally relevant reprogramming of fibroblasts. However, the lack of direct comparisons in functional barrier assays (i.e. lack of data from “negative” and “positive” control samples using primary fibroblasts (negative control) and primary vascular endothelial or smooth muscle cells (positive controls)) in all subsequent studies that use cells directly reprogrammed from young and old donors make interpretation of those data difficult. Further, the differences in permeability between barriers established with induced cells from old and young donors are very small, and may be related to small differences in integrity or health of induced cells that retain (as suggested by the transcriptomic studies) most of their fibroblast-like features. These are important concerns that need to be addressed.

We thank the reviewer for the detailed comments on our manuscript. We agree with the reviewer that an effective reprogramming protocol is fundamental to obtain meaningful data and we apologize for the absence of key data supporting this issue in the original manuscript. We have now included additional results showing that:

1) The expression of the master regulators ETV2 and MYOCD is comparable in reprogrammed cells derived from young vs. old donors (log_2_FC=0.19 for ETV2 and log_2_FC=0.03 for MYOCD comparing young and old cells; no statistical difference comparing young and old cells). This point demonstrates that the reprogramming efficiency is not affected by the age of the donor and that the observed differences between young and old cells are not due to different levels of reprogramming. Details have been included in the manuscript. We would like to highlight that permeability values were about 50% higher in presence of reprogrammed cells from old vs. young donors. This difference was consistent across multiple replicates and donors. However, we need to highlight that our microscale models do not include several components of the vascular niche, including tissue-specific cells and immune cells, which might contribute to changes in vascular functionality during aging and disease. The absence of these components might partially explain why we observed limited (~50%) differences in vascular functionality. We have included a paragraph in the manuscript to discuss this specific point.

2) The expression of most key cell identity genes for both iVECs and iSMCs is comparable in reprogrammed and primary cells. In addition, the expression of key fibroblast genes is significantly lower in both reprogrammed and primary cells compared to the fibroblast population. Details have been included in Supplementary Information (Supplementary file 2). Together, these data show that reprogrammed cells are not only expressing cell identity genes, but also downregulating well established fibroblast markers.

As pointed out by the reviewer, a “handful of vascular-specific genes might be sufficient to direct the functionality of reprogrammed cells”. However, in the original manuscript there was no direct comparison of vascular permeability among reprogrammed cells, primary VECs (positive control) and fibroblasts (negative control). We have performed additional experiments showing that the permeability of reprogrammed cells is significantly lower compared to fibroblasts, reaching values close to the permeability of primary VECs. See Figure 2I.

Although we cannot claim that reprogrammed cells are transcriptionally and functionally identical to primary VECs, these data clearly demonstrate that reprogrammed cells have acquired a “minimum set of transcriptional and functional features”, which allows to employ them to capture a fraction of the vascular changes occurring during aging and disease. We have included these new data and their discussion in the manuscript. Please note that we performed experiments to measure the barrier functionality of iVECs compared to primary VECs and fibroblasts. Indeed, fibroblasts represent an appropriate negative control for the study of vascular functionality, since they do not contribute to the formation of the inner wall of blood vessels (which is formed by VECs). However, regarding SMCs we have only performed experiments comparing iSMCs and primary SMCs. The main reason is that fibroblasts normally populate the extracellular matrix surrounding blood vessels and directly contribute to the homeostasis of VECs. Hence, a comparison including fibroblasts would have been confusing. Collected data show that the vascular permeability in presence of young iSMCs is even lower compared to primary SMCs, demonstrating that reprogramming generates functional cells directly contributing to the maintenance of the vascular barrier (potentially through paracrine secretions). See Figure 2J.

Reviewer #2:

[…] Based on the heatmap data shown in Figure 1, it is not clear whether the transcriptome of reprogrammed cells represent fully reprogrammed and functional iVECs/iSMCs. The expression data indicate that both iSMCs and iVECs express more genes in common with fibroblasts than with primary VECs or SMCs. The authors state "transition towards full mature cells." The readers will be better informed if the authors clarify what is the measurement for transcriptomics and function for partial versus fully reprogrammed cells, or present additional data that either reject or support fully mature versus partial reprogramming. It is understandable that the goal here is to induce SMCs or VECs to the point that can capture some of the age-associated changes, not necessarily generating cells that will be identical to primary cells. Still, this will be useful information to the readers.

We thank the reviewer for the useful comments on our manuscript. We have included additional data to better clarify the concept of “partial maturation” of the reprogrammed cells. First, we have analyzed the expression level of key cell identity genes for both iVECs and iSMCs and we have compared it with primary cells and fibroblasts. We have included in Supplementary Information a detailed table with these genes to provide a quantification of our concept of “partial maturation” (Supplementary file 2). For example, the expression level of KDR (an important gene defining endothelial cell identity) was 6.62 log_2_FC higher in primary VECs vs. fibroblasts and 6.38 log_2_FC higher in iVECs vs. fibroblasts. At the same time, CDH5 was upregulated in both primary cells (log_2_FC=6.7) and iVECs (log_2_FC=4) compared to fibroblasts, although at different levels. Similarly, both primary and reprogrammed SMCs upregulated key identity genes including ACTA2 and MYH10 compared to fibroblasts. However, the expression of MYH10 was lower in reprogrammed vs. primary SMCs. Finally, we would like to highlight that reprogrammed cells downregulated the expression of fibroblast-specific genes (e.g. FSP1, TWIST, SLUG), again indicating a transition towards fully mature vascular cells.

As a second step, we have performed additional experiments to compare the functionality of iVECs with primary VECs (positive control) and original fibroblasts (negative control). Our data show that the permeability of iVECs is significantly lower compared to fibroblasts, reaching values close to the permeability of primary VECs. Regarding SMCs we have only performed experiments comparing iSMCs and primary SMCs. The main reason is that fibroblasts normally populate the extracellular matrix surrounding blood vessels and directly contribute to the homeostasis of VECs. Hence, a comparison including fibroblasts would have been confusing. Collected data show that the vascular permeability in presence of young iSMCs is even lower compared to primary SMCs, demonstrating that reprogramming generates functional cells directly contributing to the maintenance of the vascular barrier (potentially through paracrine secretions).

We have included these data in the manuscript. See Figure 2I and J.

Overall, we think that these additional quantifications provide a better picture of vascular reprogramming and highlight that iVECs and iSMCs show transcriptional and functional features of VECs and SMCs, respectively, although they do not fully replicate primary cells. A discussion paragraph has been included in the manuscript.

The choice of some of the SMC markers was not clear. For example, Calponin (shown in Figure 1B) appears to be expressed in cells that do not express the reprogramming factor, MYOCD, suggesting that Calponin could be also expressed in human fibroblasts. As such, it will be informative to the readers if the authors show the same immunostaining data for Calponin in fibroblasts.

The image reported in Figure 1B showed MYOCD positive cells expressing Calponin. We noticed that a few cells can express Calponin despite the weaker MYOCD signal. We apologize if this aspect was confusing. To further clarify the expression of smooth muscle cell markers we have quantified the percentage of MYOCD positive cells co-expressing αSMA and Calponin. We have found that 70% MYOCD positive cells expressed αSMA, while 80% expressed Calponin. We have introduced these data in the manuscript.

The main focus in Figure 2 is to define age-associated changes modeled through direct reprogramming of fibroblasts of young and old age groups. It is then essential to provide experimental evidence that the comparative analyses were performed in the realm of similar reprogramming efficiencies between young and old groups. This point is particularly crucial as fibroblasts from different ages may not reprogram with the same efficiency.

We agree with the reviewer. We have provided additional data (Supplementary Information) showing that the reprogramming efficiency is comparable in cells obtained from young and old donors (log_2_FC=0.19 for ETV2 and log_2_FC=0.03 for MYOCD comparing young and old cells; no statistical difference comparing young and old cells).

It will be good to clarify whether the difference in dextran permeability between young and old cells shown in Figure 2H may be influenced by differences in reprogramming efficiency. Looking at Figure 2H, there appears to be a difference in dextran permeability, but the raw data in Figure S6 show there are very few SMA+ cells (just one red cell) in the old sample and none at the interface with iVECs, in contrast to the young sample. To further dissect the difference in permeability due to aging, not the lack of reprogrammed cells in the old samples, it will be informative to provide clear pictures showing the reprogrammed cells at the interface of the permeability assay.

We thank the reviewer for the possibility to clarify this aspect. As discussed in the previous comment, we have provided additional data showing that the reprogramming efficiency was similar in cells obtained from young and old donors. Hence, differences in permeability are not due to different reprogramming efficiency.

Regarding the distribution of iSMCs in the microscale assay, we realized that our description in the manuscript was not sufficiently clear. Indeed, iVECs and iSMCs are seeded in two separate channels, with iVECs forming a monolayer at the interface with the channel containing iSMCs (embedded in a 3D matrix). iSMCs homogeneously distribute inside the matrix channel. iSMCs from young donors tend to migrate towards the endothelial monolayer. However, the same migratory behavior is not observed with cells from old donors. Here we report a new Figure 2—figure supplement 6 showing a detail of the endothelial monolayer (VE-cadherin, white) between the pillars of the microscale model; smooth muscle actin (red) positive cells are close to the monolayer only in young donors (nuclei are shown in blue; single slice from a confocal stack).

It is important to highlight that the contribution of SMCs to the vascular barrier could be in terms of both paracrine secretion and direct support to the endothelial monolayer. Overall, IF images showed that iSMCs from old donors did not physically interact with iVECs; at the same time, differential expression analysis highlighted that iSMCs from old donors upregulated genes which might interfere with the integrity of the barrier (e.g. BMP2 which was identified through RNAseq and, at the protein level, through IF of human skin biopsies). Although we cannot separate the contribution of these two aspects, it is likely that the combination of physical support and secreted factors contributes to the observed differences in the barrier functionality in young vs. old conditions. We have included a paragraph in the Discussion section to better explain this point.

As the end of Figure 2 was currently left with the correlation studies, additional mechanistic insights into the identified DE genes will greatly signify the authors' findings, for example by either i) reducing the expression of BMP2 or PALD1 in old cells, or ii) overexpressing those aging-associated genes in young cells to pinpoint whether the identified DE genes are underlying the observed phenotypes.

We have performed additional experiments to demonstrate the functional significance of the identified genes. We thought that KD of *PALD1* and *BMP2* in primary VECs and SMCs, respectively, would have been even more relevant to clarify the contribution of these genes in the vascular functionality. Collected data showed that KD of PALD1 in VECs decreased vascular permeability compared to luciferase control (51.5±3.4% vs. 100.0±4.3%, at least N=60 independent measurements per condition in 3 biological replicates, Student’s t-test with p<0.001). On the other side, KD of BMP2 in SMCs induced a weak, but reproducible, decrease in permeability compared to luciferase control (86.3±3.8% vs. 100.0±4.3%, at least N=60 independent measurements per condition in 3 biological replicates, Student’s t-test with p=0.07).

Together, these results clearly show that *PALD1* expression by VECs is a key determinant of vascular permeability. On the other side, the contribution of BMP2, which is secreted by SMCs, is more limited. Based on this finding, it is possible that the effect of SMCs on the regulation of vascular permeability is the result of the action of multiple genes, each giving an incremental or synergistic contribution (Figure 2A includes other potential candidates). Overall, these experiments show that reprogrammed cells are a promising tool to identify potential contributors to vascular aging. Data have been included in the manuscript. See Figure 2K and L.

BMP2 was discovered to be upregulated in aging in Figure 2, but all the HGPS experiments in Figure 3 were associated with a different gene, BMP4. Based on the existing datasets, is BMP4 also upregulated in aged induced cells? Also, JAG1 is introduced in Figure 3, while there is no mention of PALD1 from Figure 2. The text will benefit from a more in-depth discussion of the link between findings of Figure 2 to Figure 3.

We thank the reviewer for the possibility to clarify this point. We did not find overlapping genes comparing young vs. old and healthy vs. HGPS cells. This is the reason why JAG1 was not mentioned in Figure 2. However, it is interesting to highlight that BMP2 (identified in Figure 2) and BMP4 (identified in Figure 3) share a 62% sequence similarity and are functionally equivalent. Hence, BMP2/4 dysregulation might underlie a common mechanism leading to vascular degeneration during physiological aging and disease. We have clarified these aspects in the Discussion section.

Reviewer #3:

Direct reprogramming of fibroblasts from young, aged, and HGPS donors into vascular endothelial cells (VECs) and smooth muscle cells (SMCs), combined with transcriptomics analyses and co-culture assays, is a novel and interesting approach to study age-related alterations in EC-SMC crosstalk and discover new potential therapeutic targets to fight vascular dysfunction in normal and premature aging. However, RNAseq results from the physiological aging-related part lacks functional validation, and RNAseq results from the HGPS-related part presents weak in vivo/ex vivo validation.

1) Regarding cells derived from normally aged donors, the authors show validation of three hits obtained from the RNAseq assays by immunofluorescence analysis of skin biopsies. However, no studies were performed to analyze the functional significance of such expression changes on endothelial permeability, as done for HGPS-related studies (e.g., using blocking antibodies or shRNA).

We agree with the reviewer that RNAseq data from the physiological aging part were not coupled with a functional validation of the identified genes. Hence, we have provided additional data showing that KD of *PALD1* and *BMP2* in VECs and SMCs, respectively, is coupled with a decrease in vascular permeability. More in detail, KD of *PALD1* in VECs decreased vascular permeability compared to luciferase control (51.5±3.4% vs. 100.0±4.3%, at least N=60 independent measurements per condition in 3 biological replicates, Student’s t-test with p<0.001). On the other side, KD of BMP2 in SMCs induced a weak, but reproducible, decrease in permeability compared to luciferase control (86.3±3.8% vs. 100.0±4.3%, at least N=60 independent measurements per condition in 3 biological replicates, Student’s t-test with p=0.07).

Together, these results clearly show that *PALD1* expression by VECs is a key determinant of vascular permeability. On the other side, the contribution of BMP2, which is secreted by SMCs, is more limited. Based on this finding, it is possible that the effect of SMCs on the regulation of vascular permeability is the result of the action of multiple genes, each giving an incremental or synergistic contribution (Figure 2A includes other potential candidates). Overall, these experiments show that reprogrammed cells are a promising tool to identify potential contributors to vascular aging. Data have been included in the manuscript. See Figure 2K and L.

2) Although the HGPS-related part of the paper includes in vitro functional studies, ex vivo and in vivo validation of the RNAseq results appears rather limited. BMP4 ELISA data in Figure 2B comparing age-matched controls and HGPS patients is very far from reaching statistical significance (p=0.4), thus making very difficult to draw conclusion. I concur with the authors that these experiments are very challenging considering the limited number of available HGPS samples, and mouse studies may help overcome this limitation. Regarding mouse studies in Figure 3C, the authors used the t-test to compare serum BMP4 levels in young, aged and HGPS groups. However, t-test is not appropriate for studies including more than 2 experimental groups. These results should be re-analyzed using ANOVA and post-hoc analysis. Given the limitation of the human studies, a more thorough validation using the mouse model would significantly contribute to strengthen the biological significance of the results. For example, it would be very informative to perform qPCR and/or immunofluorescence assays to assess BMP4 and Jagged 1 levels in mouse arteries. I also miss experiments to examine in vivo endothelial permeability and the effects of neutralizing anti-BMP4 and anti-Jagged 1 antibodies in the mouse models of normal and premature aging.

We appreciate that the reviewer concurs with us that experiments on HGPS are challenging considering the limited availability of samples and models. Based on the reviewer comments and on comments by another reviewer, we have re-analyzed data on serum from human donors by considering the aging perspective. While healthy donors show a significant decrease in BMP4 over age (t-test, p=0.04), this trend is lost in HGPS samples. This finding offers a novel way to analyze the potential contribution of BMP4 in vascular degeneration and has been included in the manuscript.

We also agree with the reviewer that a t-test was not ideal to analyze the level of BMP4 in mouse samples given the presence of 3 experimental groups. For this reason, we have performed an ANOVA test followed by Holm-Sidak test finding that HGPS samples were statistically different compared to old (p<0.001) and young (p=0.001) samples. However, there was no difference comparing old and young samples (p=0.79). We have included these data in the manuscript.

We agree with the reviewer that in vivo experiments (e.g. measurements of vascular permeability in presence of anti-BMP4 antibody) would significantly strengthen the conclusions of the manuscript. BMP4 might represent a robust biomarker of vascular damage in HGPS, with potential consequences for the development of new therapies. However, we think that these experiments might go slightly beyond the scope of this manuscript as a Tool and Resource article, which is “assessed in terms of the potential to facilitate experiments that address problems that to date have been challenging or even intractable”.

3) Progerin expression should confirmed in HGPS-derived cells.

We have provided additional information about the source of HGPS cells. All HGPS cell lines were obtained from the Progeria Research Foundation and screened for mutation in the LMNA gene, as certified by the Progeria Research Foundation (https://www.progeriaresearch.org/wp-content/uploads/2019/11/PRF-AVAILABLE-CELL-LINES^-1^.pdf).

4) Several references appear to be incorrect in the introductory paragraph in the section of Results focused on HGPS. The authors state: "In particular, HGPS is characterized by dysfunctional SMCs, which promote vascular calcification (Wei et al., 2018), hypertension (Miriyala et al., 2006) and loss of vascular barrier functionality (Helbing et al., 2017)". The cited papers address the role of BMPs in vascular calcification, hypertension and loss of vascular barrier functionality, but I believe none of them include studies with either HGPS patients or HGPS experimental models. The same comment regarding the studies by Wei et al., 2018 and Miriyala et al., 2006 applies to the Discussion: "The main cardiovascular complications observed in HGPS patients include loss of SMC coverage of blood vessels (Varga, et al., 2006), abnormal vascular calcification (Wei, et al., 2018) and hypertension (Miriyala, et al., 2006)." Also note that all results in Varga et al., 2006 were derived from a mouse model of HGPS, not from HGPS patients, therefore references for SMC loss in patients should be cited instead of Varga et al., 2006 (or 1-2 review articles).

We apologize for the incorrect references. We have modified the text accordingly.

5) Figure 1A, B: Quantitative data should be presented (eg. % aSMA/Myocardin-immunoreactive and % Calponin/Myocardin-immunoreactive cells).

We have quantified the percentage of MYOCD positive cells co-expressing αSMA and Calponin. We have found that 70% MYOCD positive cells expressed αSMA, while 80% expressed Calponin. We have introduced these data in the manuscript.

6) Figure 1D: The authors state that iSMCs contribute to the formation of microvascular networks in 3D fibrin matrices (Figure 1D), but no control without iSMCs is shown, which is needed to ascertain if iSMC promote this process.

We apologize for the misunderstanding. We wanted to highlight that iSMCs physically interact with VECs and partially wrap segments of capillary structures. Based on the reviewer comment, we have re-analyzed data generated in our lab regarding the area fraction covered by VECs (vascular structures) in presence of fibroblasts (co-culture model; fibroblasts are considered an optimal source of growth factors to promote the formation of in vitro vascular networks). The same culture conditions (cell culture medium, 3D matrix, cell densities, time) were applied in the co-culture model with iSMCs. We have then quantified the area fraction covered by VECs in presence of fibroblasts or iSMCs considering regions of interests of the same dimensions. Our data show that there is not a statistical difference (t-test with p=0.61) when VECs are co-cultured with fibroblasts or iSMCs, supporting the idea that iSMCs contribute to the formation of vascular networks in vitro (Author response image 1).

**Author response image 1.** Quantification of the vascular area fraction in 3D matrices embedding a co-culture of VECs with fibroblasts or iSMCs (Student’s t-test with p=0.61).

7) The images in Figure 2E and 2F do not seem to be very representative according to the quantitative data shown in Figure 2C and 2D (massive upregulation in images, but only 2-fold induction in the graphs).

We apologize, the images were selected to be representative for the differential expression of selected proteins. Image quantification is based on thresholded images (binarized) where low intensity pixels (which can be barely detected by eyes in the images) might be amplified resulting in the lower difference reported in the graphs.

8) Figure 2G-J: To confirm that age-related increased permeability is due to compromised VEC junctions, confocal microscopy experiments should be performed in young and old VECs to visualize junctions within neighbouring cells, ideally in monoculture and in co-culture with young and old SMCs.

We have included additional data showing the expression of Y658 VE-Cadherin (marker of cell-cell junction de-stabilization) in iVECs derived from young and old donors. Quantification of Y658 area fraction showed a significant over-expression of this marker in iVECs from old donors, complementing data on vascular permeability reported in Figure 2—figure supplement 5.

9) Figure 3D, F and G: The experiments include 3 groups, therefore t-test is not adequate. Results should be re-analyzed using ANOVA and post-hoc analysis.

We have re-analyzed the data using ANOVA test. Details are reported below.

Figure 3D: ANOVA with Holm-Sidak test gives p<0.001 for all comparisons

Figure 3F: ANOVA with Holm-Sidak test gives p<0.001 for all comparisons

Figure 3G: ANOVA with Holm-Sidak test gives p=0.01 for control vs. Jagged1; p=0.043 for control vs. BMP4; p=0.897 for BMP4 vs. Jagged1

Results have been included in the manuscript.

10) Figure 3H-J: Quantitative results should be shown.

Quantification for Figure 3H-J was already present in Figure 3G.

11) Figure 2—figure supplement 3: Quantitative results of GSTM1 expression in young versus old ECs should be shown (as done for BMNP2 and PALD1 in Figure 2C and 2D, respectively.

Quantification for *GSTM1* has been included in Supplementary Information.

[Editors’ note: what follows is the authors’ response to the second round of review.]

Essential revision requirements:

The authors addressed most comments appropriately with new experimental results or new statistical analyses. However, the question of similar reprogramming efficiency between young and old samples (an essential point to the manuscript), was still not fully answered. In the revised manuscript, the authors compared the expression of ETV2 and MYOCD and found no statistical difference. However, these are comparisons of the expression level of reprogramming factors, not the reflection of similar reprogramming efficiency between young and old samples – that is, whether the reprogrammed state into iVECs or iSMC as an output, is similar between young and old samples. This is a central point that needs to be addressed.

We realize that our answer regarding the question about similar reprogramming efficiency between young and old samples by showing expression of the reprogramming factors ETV2 and MYOCD did not fully satisfied the reviewers. We now provide evidence showing that the reprogramming efficiencies are similar. We have performed additional analyses to show that the expression of key cell identity genes did not change comparing cells derived from young and old donors, considering both induced endothelial cells (iVECs) and induced smooth muscle cells (iSMCs). We have included a Supplementary File 3 reporting the log_2_FoldChange (FC) and the False Discovery Rate (FDR) for these genes. As you can see, the expression of these genes is not statistically different comparing young and old induced cells.

In addition, we have generated heat-maps to better visualize how the expression of all these genes is not affected by the age group. As a reference, we have also included fibroblasts derived from young and old donors. These heat-maps have been included as Supplementary Figure (Z-score: ±1) (Figure 2—figure supplement 1). If you think that these additional images might better fit in the main text, we are open to include them in Figure 1.

The main text has now been modified considering these details. We think that these data together with the expression of ETV2 and MYOCD will clarify the concept that differences between young and old reprogrammed cells are not dependent on their identity.

We have now changed the way supplementary figures, videos and tables are cited. Moreover, supplementary methods and legends are now included in the main text. The Key Resources Table has now been included at the beginning of the Materials and methods Section. Regarding cell lines, relevant information have been added to the Supplementary Materials and methods section. In addition, we have provided references in the Key Resources Table to clarify that all cell lines were characterized for cell identity and tested negative for mycoplasma. We have provided a more detailed ethics statement, which is reviewed and approved yearly by the IRB. Regarding plasma from mice, we would like to highlight that we did not perform any kind of animal experiment. Plasma was obtained from Prof. Belmonte at Salk Institute. Relevant sequencing data are uploaded and publicly available in GEO (accession number GSE140898) and analyzed data are also included in Source Data 1, Source Data 2 and Source Code 1.
